# Chitosan‐Gelatin‐EGCG Nanoparticle‐Meditated LncRNA TMEM44‐AS1 Silencing to Activate the P53 Signaling Pathway for the Synergistic Reversal of 5‐FU Resistance in Gastric Cancer

**DOI:** 10.1002/advs.202105077

**Published:** 2022-06-19

**Authors:** Mi Zhou, Jiaqi Dong, Junqing Huang, Wen Ye, Zhousan Zheng, Kangbo Huang, Yihui Pan, Junjie Cen, Yanping Liang, Guannan Shu, Sheng Ye, Xuanxuan Lu, Jiaxing Zhang

**Affiliations:** ^1^ Department of Oncology The First Affiliated Hospital of Sun Yat‐sen University No. 58, Zhongshan road II Guangzhou 510080 P. R. China; ^2^ Guangzhou Key Laboratory of Formula‐Pattern of Traditional Chinese Medicine Formula‐Pattern Research Center School of Traditional Chinese Medicine Jinan University Guangzhou 510632 P. R. China; ^3^ Department of Urology The First Affiliated Hospital of Sun Yat‐sen University No. 58, Zhongshan road II Guangzhou 510080 P. R. China; ^4^ Department of Food Science and Engineering Jinan University Guangzhou 510632 P. R. China

**Keywords:** 5‐FU resistance, gastric cancer, lncRNAs, nanoparticles, p53 signaling pathway

## Abstract

Chemoresistance is one of the leading causes of therapeutic failure in gastric cancer (GC) treatment. Recent studies have shown lncRNAs play pivotal roles in regulating GC chemoresistance. Nanocarriers delivery of small interfering RNAs (siRNAs) to silence cancer‐related genes has become a novel approach to cancer treatment research. However, finding target genes and developing nanosystems capable of selectively delivering siRNAs for disease treatment remains a challenge. In this study, a novel lncRNA TMEM44‐AS1 that is related to 5‐FU resistance is identified. TMEM44‐AS1 has the ability to bind to and sponge miR‐2355‐5p, resulting in the upregulated PPP1R13L expression and P53 pathway inhibition. Next, a new nanocarrier called chitosan‐gelatin‐EGCG (CGE) is developed, which has a higher gene silencing efficiency than lipo2000, to aid in the delivery of a si‐TMEM44‐AS1 can efficiently silence TMEM44‐AS1 expression to synergistically reverse 5‐FU resistance in GC, leading to a markedly enhanced 5‐FU therapeutic effect in a xenograft mouse model of GC. These findings indicate that TMEM44‐AS1 may estimate 5‐FU therapy outcome among GC cases, and that systemic si‐TMEM44‐AS1 delivery combined with 5‐FU therapy is significant in the treatment of patients with recurrent GC.

## Graphical abstract

5‐FU resistance is a severe clinical challenge in gastric cancer treatment. In an in vivo 5‐FU‐resistant gastric cancer model, TMEM44‐AS1 is identified as a key factor in inducing 5‐FU resistance by promoting proliferation and inhibiting apoptosis. In addition, novel chitosan‐gelatin‐EGCG nanoparticles are developed to deliver si‐TMEM44‐AS1 into an in vivo xenograft model and succeed reversing 5‐FU resistance.

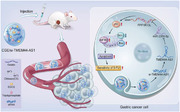

## 1 Introduction

Gastric cancer (GC) takes the fifth and third places in terms of morbidity and cancer‐related death worldwide. In China, GC morbidity and mortality rates are higher than the global average.^[^ ^1^ ^]^ The mainstay of treatment for GC patients is surgery combined with chemotherapy.^[^ ^2^ ^]^ Generally, 5‐fluorouracil (5‐FU)‐based regimens are commonly considered as the first‐line chemotherapeutic options;^[^ ^3^ ^]^ however, intrinsic or acquired resistance to 5‐FU among GC patients limits its therapeutic efficacy, leading to treatment failure. The mechanisms involved in 5‐FU resistance in GC are only partially understood and comprehensive knowledge of the molecules and processes underlying 5‐FU‐related resistance is vital if the innovative targets and strategies of therapy that allow for the improvement of chemotherapy against GC are to be developed.

Long noncoding RNAs (lncRNAs) are noncoding RNA molecules over 200 nucleotides (nt) in length. They can both influence gene expression and serve as biomarkers in diverse biological and pathophysiological contexts, highlighting their potential utility as therapeutic targets.^[^ ^4^ ^]^ Numerous researches have approved that lncRNAs play pivotal roles as early prognostic and therapeutic biomarkers in GC, as well as in the chemoresistance of this cancer.^[^ ^5^ ^]^ LncRNAs can play a role as competitive endogenous RNAs (ceRNAs), which compete against miRNAs for target mRNA binding, resulting in depression of target mRNA.^[^ ^6^ ^]^ A great amount of lncRNAs and miRNAs have been recognized as being associated with GC progression and chemoresistance.^[^ ^7^ ^]^ For instance, the lncRNA MT1JP modulates *FBXW7* level through sponging miR‐92a‐3p, thereby influencing GC progression,^[^ ^8^ ^]^ while lncRNA MALAT1 reportedly regulates autophagy‐associated chemoresistance by modulating ATG12 expression via miR‐23b‐3p.^[^ ^9^ ^]^ These observations highlight the importance of identifying other lncRNA molecules that mediate drug resistance and developing the innovative targets and strategies for the improvement of chemotherapy against GC.

Small interfering RNAs (siRNAs) may decrease target gene levels within cancer cells,^[^ ^10^ ^]^ and using nanocarriers to deliver siRNAs to silence cancer‐related genes has become a new approach to cancer treatment research.^[^ ^11^ ^]^ Although several potent, specific, and biocompatible nanoparticle siRNA delivery vehicles are already available,^[^ ^12^ ^]^ developing nanosystems that can selectively deliver siRNAs for disease treatment remains a challenge. Chitosans are natural polymers consisting of partially acetylated (1‐4)‐2‐amino‐2‐deoxy‐d‐glucan. As the biocompatible and biodegradable material, chitosan is suitable in vivo via intravenous or intraperitoneal administration.^[^ ^13^ ^]^ Chitosan‐derived nanoparticles have shown great potential for use in drug delivery.^[^ ^14^ ^]^ Moreover, because it has a positive charge under slightly acidic conditions, chitosan can form complexes with siRNAs through electrostatic interactions, characteristics that favor its use in gene therapy.^[^ ^15^ ^]^ Despite these advantageous properties, further modifications are needed to render it an ideal gene delivery vehicle, including improving its poor water solubility at physiological pH. Gelatin is a protein that can be extracted in the partial hydrolysis of collagen. Its biosafety has been validated in vivo applications as biomaterials or drugs.^[^ ^16^ ^]^ The gelatin delivery system has shown effective controlled release ability for proteins and drugs.^[^ ^17^ ^]^ With low cytotoxicity and antigenicity, it is also an attractive vehicle for controlled gene release.^[^ ^18^ ^]^ Nevertheless, the gene delivery efficiency of natural gelatin is poor owing to its loose structure and low charge density. Complexing nanoparticles with other polymers can enhance colloid stability via steric repulsion, thereby increasing their stability in the high ionic strength conditions of the biological environment.^[^ ^19^ ^]^ Accordingly, in this study, we complexed gelatin with positively charged chitosan, generating crosslinked gelatin/chitosan nanoparticles. These complexed nanoparticles would be expected to overcome the limitations associated with the original gelatin and chitosan nanoparticles, promoting their solubility and stability and thereby increasing the efficiency of delivery of intravenously administered siRNA.

In this study, HGC‐27 and MKN‐45 cells were first transplanted in nude mice which were later treated with 0.9%NaCl or 5‐FU to construct 5‐FU sensitive or resistant cell lines. Then, according lncRNA sequencing analysis, we identified lncRNA TMEM44‐AS1 as an important player in 5‐FU resistance in GC. Further investigation demonstrated that TMEM44‐AS1 can be a sponge for miR‐2355‐5p, leading to the upregulating of PPP1R13L expression and the inhibition of the P53 signaling pathway, and further indicating that TMEM44‐AS1 was the candidate prognostic marker and potential therapeutic target in 5‐FU‐resistant GC patients. Taking advantage of this regulatory mechanism, we generated chitosan‐gelatin‐epigallocatechin gallate (CGE) nanoparticles and used them to deliver siRNA targeting TMEM44‐AS1 (si‐TMEM44‐AS1) aiming to reverse 5‐FU resistance in GC. The results showed that compared with naked siRNA, the CGE‐encapsulated siRNA has a better stability in serum, and was easier to be absorbed and accumulated by GC cells. The CGE nanoparticles mediated delivery system of this study is a both simple and effective approach for inhibiting 5‐FU tolerance within GC, which may have potential for developing the new treatment method of 5‐FU‐resistant GC.

## 2 Results

### 2.1 TMEM44‐AS1 Is Upregulated in 5‐FU‐Resistant GC Cell Lines

**Figure 1:**
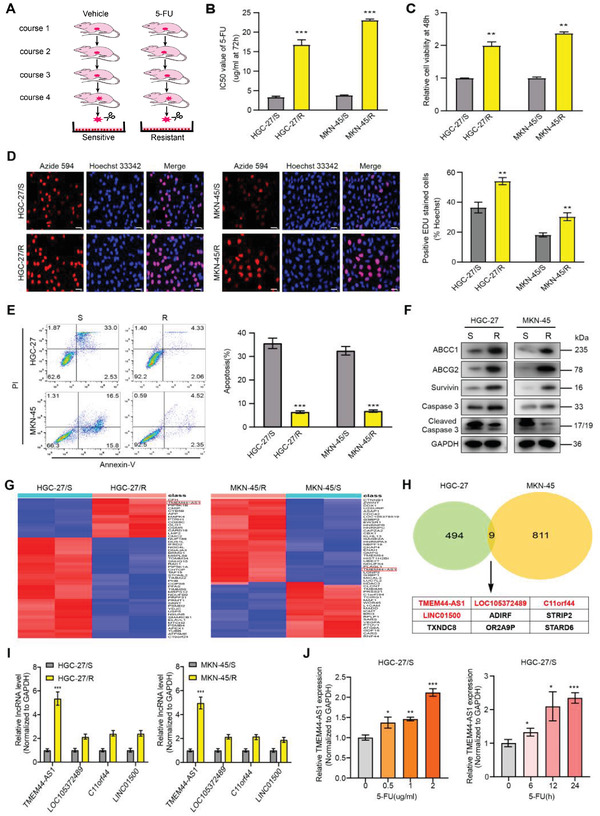
TMEM44‐AS1 is upregulated in 5‐FU‐resistant GC cell lines. A) Sketch map of 5‐FU‐resistant cell line construction. B) The IC50 value for 5‐FU was detected for both sensitive and 5‐FU‐resistant cells through CCK‐8 assay. C) After 5‐FU treatment (5 µg mL^−1^) for 48 h, the 5‐FU‐resistant and sensitive cell viability was analyzed through CCK‐8 assay. D) 5 µg mL^−1^ 5‐FU was used to treat cells for a 48 h period, following which proliferative ability of both 5‐FU‐resistant and sensitive cells was assessed through EdU assay. Scale bar, 20 µm. E) 5 µg mL^−1^ 5‐FU was used to treat cells for a 48 h period, and then we measured cell apoptosis rate for both sensitive and resistant cells through flow cytometry. F) Western blot analysis of the expression of ABCC1, ABCG2, Survivin, Caspase3, and cleaved caspase‐3 after 5 µg mL^−1^ 5‐FU treatment for 48 h. G) The heatmap shows the 50 most significantly upregulated and downregulated RNAs in MKN‐45/R and HGC‐27/R cells when relative to their respective sensitive cells as determined by transcriptome sequencing. H) Venn plot showing two data sets. Overlapped RNAs upon |log2 (fold change, FC)| ≥1 and *P* ≤ 0.05 were selected. I) RT‐qPCR analysis on expression levels of overlapping lncRNAs. J) 5‐FU treatment could induce upregulation of TMEM44‐AS1 in sensitive cell lines. Data are mean ± SD, *n* = 3. *P < 0.05, **P < 0.01, ***P < 0.001.

To establish the 5‐FU‐resistant GC cells, the HGC‐27 and MKN‐45 cell lines were transplanted in each nude mouse, and then intraperitoneally injected with 10 mg kg^−1^ 5‐FU or 0.9%NaCl according to description in the Experimental Section (**Figure** **1**A). GC xenografts receiving four 5‐FU cycles showed low 5‐FU response (Figure S1, Supporting Information). We obtained 5‐FU sensitive cell lines (HGC‐27/S and MKN‐45/S) and 5‐FU resistance cell lines (HGC‐27/R and MKN‐45/R) by primary culture of 5‐FU sensitive and resistance tumor samples, respectively. Their reactivity to the 5‐FU was then verified. According to Figure 1b, the 5‐FU IC50 values substantially increased within the resistant cell sublines compared with respective sensitive lines. Furthermore, when cells were treated using 5 µg mL^−1^ 5‐FU for a 48‐h period, the viability of cells from both 5‐FU‐resistant lines was markedly improved compared with that in the sensitive cell lines (Figure 1C). We then used EdU and CCK‐8 assays for evaluating cell proliferative ability from both cell lines after 5‐FU treatment (5 µg mL^−1^, 48 h). As a result, the resistant cells had greater proliferative ability compared with the respective sensitive cells (Figure 1D and Figure S2, Supporting Information). Flow cytometric analysis further indicated the markedly reduced cell apoptosis of resistant cells after 5‐FU therapy (Figure 1E). We further assessed the expression levels of multidrug resistance‐ and apoptosis‐related proteins in 5‐FU‐sensitive and 5‐FU‐resistant cell lines by western blotting (WB). We found that the protein expression levels of ABCC1, ABCG2, Survivin, and Caspase3 were upregulated in the 5‐FU‐resistant cell sublines, whereas that the level of cleaved caspase‐3 was downregulated (Figure 1F). To identify lncRNAs that might potentially influence GC cell resistance to 5‐FU, this study used transcriptome sequencing for analyzing and comparing lncRNA expression profiles between 5‐FU‐resistant and sensitive cells (Figure 1G). As shown in Figure 1H, four lncRNAs were upregulated in both HGC‐27/R and MKN‐45/R sublines, namely, TMEM44‐AS1, LOC105372489, C11orf44, and LINC01500. RT‐qPCR analysis further confirmed that only TMEM44‐AS1 expression was the most significantly upregulated in HGC‐27/R as well as MKN‐45/R cells relative to respective 5‐FU sensitive cell controls (Figure 1I). Therefore, we also assessed whether 5‐FU treatment could induce TMEM44‐AS1 upregulation in sensitive cell lines. As shown in Figure 1J and Figure S3 (Supporting Information), TMEM44‐AS1 expression was also upregulated within HGC‐27/S and MKN‐45/S cells depending on the dose and time. Taken together, these results clearly indicated that high TMEM44‐AS1 expression was related to 5‐FU resistance of GC cells.

### 2.2 TMEM44‐AS1 Mainly Existed in Cytoplasm in GC Cells and Was Indicative of Poor Outcome

**Figure 2:**
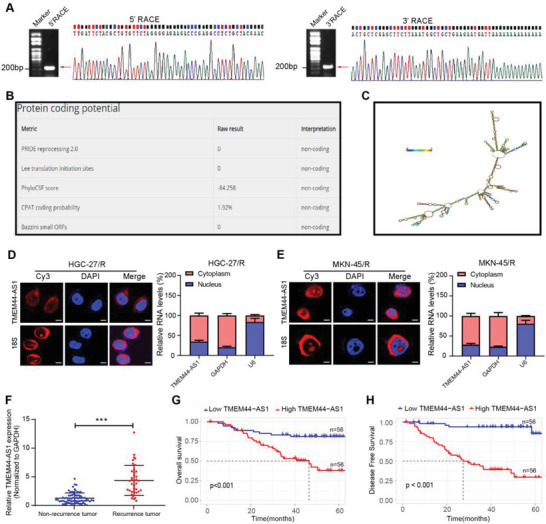
TMEM44‐AS1 mainly existed in cytoplasm in GC cells and was indicative of poor outcome. A) PCR products from rapid amplification of cDNA ends (RACE). The products (indicated by arrows) of 5′ and 3′‐RACE were obtained by nested PCR and then sequenced. 5′‐ or 3′‐sequences of the TMEM44‐AS1 transcript are marked. B) Prediction of protein coding potential of TMEM44‐AS1 from LNCipedia (http://www.lncipedia.org). C) Diagram of TMEM44‐AS1 secondary structure prediction (RNAfold web server). D,E) Fluorescence in situ hybridization (FISH) and subcellular fractionation assays showing the main localization of TMEM44‐AS1 within GC cell cytoplasm. 18S/GAPDH and U6 served as cytoplasmic and nuclear controls, respectively. Scale bar, 10 µm. F) Comparison of differences in TMEM44‐AS1 expression levels within GC tissues between non‐recurrence (*n* = 74) and recurrence (*n* = 38) cases. G) OS and H) DFS in GC cases (*n* = 112) showing different levels of TMEM44‐AS1 expression in their tumors. Data are mean ± SD, *n* = 3. *P < 0.05, **P < 0.01, ***P < 0.001.

TMEM44‐AS1 is located on Chromosome 3q29 and we determined the full‐length sequence of TMEM44‐AS1 (1433 bp) in GC cells through 5'‐ and 3'‐rapid amplification of cDNA ends (RACE) (**Figure** **2**A). Through predictions in the online database (LNCipedia [http://www.lncipedia.org]), we found that TMEM44‐AS1 has a very low coding potential (Figure 2B). The structure of TMEM44‐AS1 is depicted in Figure 2C. As suggested by subcellular fractionation and fluorescence in situ hybridization (FISH) assays, TMEM44‐AS1 mainly existed in cytoplasm in GC cells (Figure 2D,E). Next, we used RT‐qPCR for examining TMEM44‐AS1 levels in 112 stage II–III GC cases receiving surgery plus adjuvant chemotherapy (see the Experimental Section). At follow‐up, 38 patients exhibited tumor recurrence or metastasis. We found that TMEM44‐AS1 expression remarkably increased among cases developing distant metastasis (DM) or local relapse when compared with those of patients without tumor recurrence or metastasis (Figure 2F). Importantly, high TMEM44‐AS1 expression was identified as being positively related to dismal OS (Figure 2G) and DFS (Figure 2H). For better determining whether TMEM44‐AS1 level was of certain prognostic significance, RT‐qPCR was conducted on 60 pretreated samples collected in GC cases at stage IV receiving palliative chemotherapy. Of them, no one achieved complete response (CR), 30 attained partial response (PR), 17 had stable disease (SD), whereas 13 had progressive disease (PD) when being evaluated. Moreover, TMEM44‐AS1 was negatively correlated with chemotherapy responses in our enrolled cases, i.e., high TMEM44‐AS1 expression was detected more frequently in the SD + PD subset (23/30, 76.7%) than in the CR + PR subset (10/30, 33.3%) (*P* < 0.001, Table S3, Supporting Information). Additionally, we conducted RT‐qPCR assay to test the expression level of TMEM44‐AS1 in GC cell lines and normal epithelial cells and found that TMEM44‐AS1 upregulated in most GC cell lines compared to normal cells (Figure S4A, Supporting Information). We further analyzed transcriptome data from The Cancer Genome Atlas (TCGA) database and found that TMEM44‐AS1 was not only upregulated in the GC cells and tumor samples but was also correlated with poor DFS in GC samples from TCGA database (Figure S4B, Supporting Information). Analysis of TCGA data also revealed that TMEM44‐AS1 upregulation was frequently detected in tumors other than GC (Figure S4C, Supporting Information). Combined, these results indicated that high TMEM44‐AS1 expression was related to resistance of GC patients to 5‐FU, and that the evaluation of TMEM44‐AS1 expression contributed to predicting response to 5‐FU therapy among GC cases.

### 2.3 TMEM44‐AS1 Induced GC Resistance to 5‐FU by Suppressing P53 Pathway

**Figure 3:**
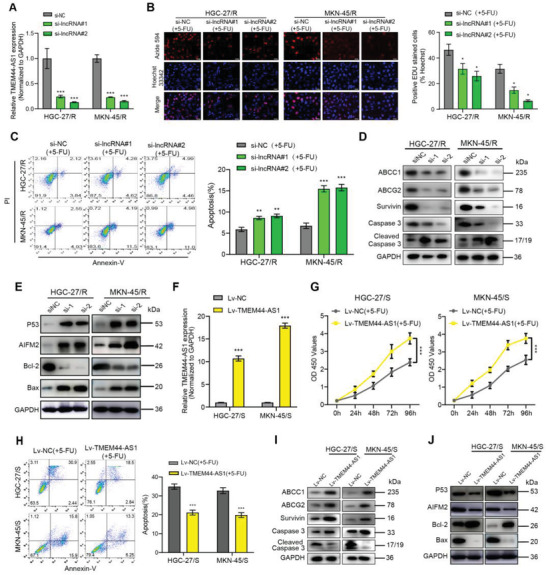
TMEM44‐AS1 induced GC resistance to 5‐FU by suppressing the P53 pathway. A) TMEM44‐AS1 silencing efficiency was measured through RT‐qPCR following si‐TMEM44‐AS1#1 and si‐TMEM44‐AS1#2 infection. B) Proliferative abilities of HGC‐27/R and MKN‐45/R cells subject to TMEM44‐AS1 siRNAs or si‐NC transfection were measured through EdU assay. Scale bar, 20 µm. C) The cell apoptosis rate for both HGC‐27/R and MKN‐45/R cells was assessed through flow cytometry. D) ABCC1, ABCG2, Survivin, Caspase3, cleaved caspase‐3, and GAPDH protein expression was detected through WB in two 5‐FU‐resistant cell lines following transfection with TMEM44‐AS1 siRNAs or si‐NC. E) P53, AIFM2, Bcl‐2, Bax, and GAPDH protein expression in HGC‐27/R and MKN‐45/R cells subject to TMEM44‐AS1 siRNAs or si‐NC treatment. F) RT‐qPCR was used to detect the overexpression effect of TMEM44‐AS1 after infection with Lv‐TMEM44‐AS1. G) 5‐FU (5 *μ*g mL^−1^) was added to cell medium, cell proliferation ability of HGC‐27/S and MKN‐45/S transfected with Lv‐TMEM44‐AS1 or Lv‐NC was detected by CCK8 assay. The relative proliferative rates at different time points were normalized to 0 h. H) Cell apoptosis rate was detected for both sensitive cells by flow cytometry analysis. I) Protein expression levels of ABCC1 and ABCG2, Survivin, Caspase3, cleaved caspase3, and GAPDH were detected by western blotting after both sensitive cell lines were transfected with Lv‐TMEM44‐AS1 or Lv‐NC. J) Protein expression levels of P53, AIFM2, Bcl‐2, Bax, and GAPDH in HGC‐27/S and MKN‐45/S cells treated with Lv‐TMEM44‐AS1 or Lv‐NC. 5 µg mL^−1^ 5‐FU was used to treat cells for a 48‐h period before EdU assay, CCK8 assay, flow cytometric analysis, and protein extraction. Data are mean ± SD, *n* = 3. *P < 0.05, **P < 0.01, ***P < 0.001.

Having validated that TMEM44‐AS1 showed high expression within GC cells with 5‐FU resistance, we next explored the associated underlying mechanisms. For investigating TMEM44‐AS1's biological role, two siRNAs were constructed, both of which induced a significant downregulation of TMEM44‐AS1 expression in HGC‐27/R and MKN‐45/R cells (**Figure** **3**A). Next, we used EdU and CCK‐8 assays for comparing proliferative ability between 5‐FU‐resistant cells expressing si‐TMEM44‐AS1 and si‐NC (negative control) after 5‐FU treatment. The results showed that, compared with controls, TMEM44‐AS1 knockdown remarkably declined HGC‐27/R and MKN‐45/R cell proliferation (Figure 3B and Figure S5, Supporting Information). Moreover, flow cytometric analysis indicated that the cell apoptosis rate was markedly increased when TMEM44‐AS1 was downregulated within GC cells with 5‐FU resistance (Figure 3C). We subsequently evaluated multidrug resistance‐ and apoptosis‐related protein levels within cells with 5‐FU resistance following TMEM44‐AS1 knockdown and found that the expression of multidrug resistance‐related proteins was suppressed with TMEM44‐AS1 depletion, whereas that of apoptosis‐related proteins was enhanced (Figure 3D). According to KEGG pathway enrichment, the TMEM44‐AS1 expressing, protein‐coding genes were mainly enriched into P53 signaling pathway (Figure S6, Supporting Information). Then we undertook a bioinformatics analysis to visualize genes in the P53 pathway in our sequencing results. Interestingly, direct P53 targets associated with apoptosis, such as PIDD1, BAX, SIVA1, EI24, SHISA5, AIFM2, and IGFBP3, were downregulated (Figure S7, Supporting Information), suggesting that TMEM44‐AS1 may promote tumor chemoresistance by inhibiting the P53 signaling pathway. To test this possibility, we used western blotting to measure the changes occurring in the expression of P53 signaling pathway‐related genes in siRNAs‐transfected HGC‐27/R as well as MKN‐45/R cell line. Knockdown of TMEM44‐AS1 led to altered expression of P53 and P53 target genes (Figure 3E). We also overexpressed TMEM44‐AS1 in sensitive HGC‐27 and MKN‐45 cells by transducing lentiviral vectors containing TMEM44‐AS1 sequences (Figure 3F) and treated the transduced cells with 5‐FU (5 µg mL^−1^) for 48 h. Consistent with the findings from the knockdown experiments, cell proliferation was increased (Figure 3G and Figure S8, Supporting Information) and the rate of cell apoptosis decreased with TMEM44‐AS1 overexpression (Figure 3H). We also measured the expression levels of multidrug resistance‐, apoptosis‐, and P53 pathway‐related proteins and obtained results consistent with our previous findings (Figure 3I,J). These data suggested that TMEM44‐AS1 may inhibit apoptosis by inhibiting the P53 signaling pathway, and further indicated that TMEM44‐AS1 may be a critical regulator of 5‐FU resistance.

### 2.4 miR‐2355‐5p Is a Target of TMEM44‐AS1 in GC

**Figure 4:**
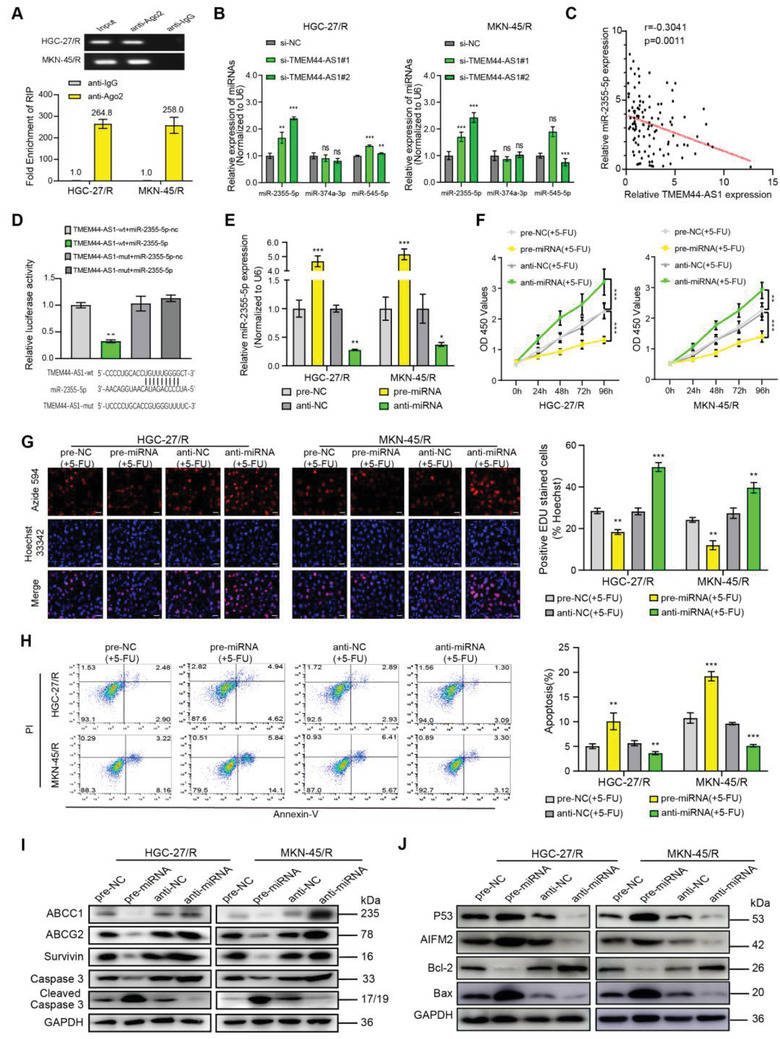
MiR‐2355‐5p is a target of TMEM44‐AS1 in gastric cancer. A) RIP experiments were performed using the Ago2 antibody, and specific primers were used to detect the enrichment of TMEM44‐AS1. B) RT‐qPCR was used to detect the potential target miRNA of TMEM44‐AS1 after knocking down TMEM44‐AS1. C) Linear regression analysis was done to each individual TMEM44‐AS1 and miR‐2355‐5p expression, *r* = −0.3041, *p* = 0.0011. D) Dual‐luciferase gene reporter assays were employed to assess the binding sites of TMEM44‐AS1 and miR‐2355‐5p. The relative luciferase activities were restrained in the HEK‐293 T cells co‐transfected with TMEM44‐AS1‐wt and miR‐2355‐5p. E) RT‐qPCR was used to detect the transfection efficiency of pre‐miR‐2355‐5p and anti‐miR‐2355‐5p. F) 5‐FU (5 *μ*g mL^−1^) was added to cell medium, the proliferative abilities of HGC‐27/R and MKN‐45/R cells subject to pre‐NC, pre‐miR‐2355‐5p, anti‐miR‐2355‐5p, or anti‐NC transfection were measured through CCK‐8 assay. Proliferation rates at diverse time periods were calculated relative to that at 0 h. G) Proliferative abilities of HGC‐27/R and MKN‐45/R cells subject to pre‐NC, pre‐miR‐2355‐5p, anti‐miR‐ 2355‐5p, or anti‐NC transfection were analyzed through EdU assay. Scale bar, 20 µm. H) The cell apoptosis rates were determined for the two 5‐FU‐resistant cells through flow cytometry. I) ABCC1, ABCG2, Survivin, Caspase3, cleaved caspase‐3, and GAPDH protein expression was measured in both 5‐ FU‐resistant lines by western blotting after transfection with pre‐NC, pre‐miR‐2355‐5p, anti‐miR‐2355‐5p, or anti‐NC. J) P53, AIFM2, Bcl‐ 2, Bax, and GAPDH protein levels in HGC‐27/R and MKN‐45/R cells treated with pre‐miR‐2355‐5p, pre‐NC, anti‐miR‐2355‐5p, or anti‐NC. 5 µg mL^−1^ 5‐FU was used to treat cells for a 48‐h period before EdU assay, flow cytometric analysis, and protein extraction. Data are mean ± SD, *n* = 3. *P < 0.05, **P < 0.01, ***P < 0.001.

As previously mentioned, TMEM44‐AS1 mainly existed in cytoplasm in GC cells. Because several studies have reported that lncRNAs in the cytoplasm sponge miRNAs and modulate miRNA expression, we next examined the role of TMEM44‐AS1 in sponging miRNA. MiRNAs assemble into miRNA ribonucleoprotein complexes (miRNPs) that include AGO2, the key part in RNA‐induced silencing complex.^[^ ^20^ ^]^ Consequently, RNA immunoprecipitation (RIP) assay was carried out using AGO2, and we found that TMEM44‐AS1 was enriched with AGO2 in the two cell types with resistance to 5‐FU (**Figure** **4**A). Next, this work used the Encyclopedia of RNA Interactomes (ENCORI, http://starbase.sysu.edu.cn/index.php) database for predicting miRNAs possibly regulated by TMEM44‐AS1. We identified three miRNAs—miR‐2355‐5p, miR‐374a‐3p, and miR‐545‐5p—as being potential TMEM44‐AS1 targets, and assessed whether the levels of these three miRNAs were altered following TMEM44‐AS1 knockdown. As a result, miR‐2355‐5p showed the highest upregulation when TMEM44‐AS1 expression was depleted (Figure 4B). Furthermore, we observed that miR‐2355‐5p level was in indirect proportion to TMEM44‐AS1 level within GC cells and the samples in the 112 GC cases (Figure 4C and Figure S9, Supporting Information). This study conducted dual‐luciferase gene reporter assays for assessing whether TMEM44‐AS1 and miR‐2355‐5p interacted. We found that miR‐2355‐5p‐transfected TMEM44‐AS1‐WT expressing cells had evidently decreased luciferase activity (Figure 4D). Having determined miR‐2355‐5p expression in GC, we next individually transfected miR‐2355‐5p‐antagomiR (anti‐miR‐2355‐5p) and miR‐2355‐5p‐agomiR (pre‐miR‐2355‐5p) into cells with 5‐FU resistance (Figure 4E) and assessed impacts on TMEM44‐AS1 expression (Figure S10, Supporting Information). The results showed that, compared with controls, TMEM44‐AS1 expression was downregulated in cells with miR‐2355‐5p overexpression and upregulated within miR‐2355‐5p depleted cells. Subsequently, we examined the impacts of altered miR‐2355‐5p expression on 5‐FU‐resistant cell proliferative and apoptotic ability. According to Figure 4F,G, proliferation was markedly reduced in miR‐2355‐5p‐overexpressing cells, and remarkably elevated within miR‐2355‐5p‐depleted cells. Flow cytometric analysis indicated that the rate of apoptosis was increased with miR‐2355‐5p upregulation but decreased with miR‐2355‐5p downregulation (Figure 4H). Based on Western blot results, miR‐2355‐5p upregulation suppressed the expression levels of ABCC1, ABCG2, Survivin, Caspase3, and Bcl‐2 and activated the expression levels of cleaved caspase‐3, P53, AIFM2, and Bax; the opposite effects were seen with miR‐2355‐5p silencing (Figure 4I,J). These results suggested that TMEM44‐AS1 can bind to miR‐2355‐5p as a sponge.

### 2.5 miR‐2355‐5p Participates in Effects on 5‐FU Resistance Associated with TMEM44‐AS1 Knockdown in Cells with 5‐FU Resistance

**Figure 5:**
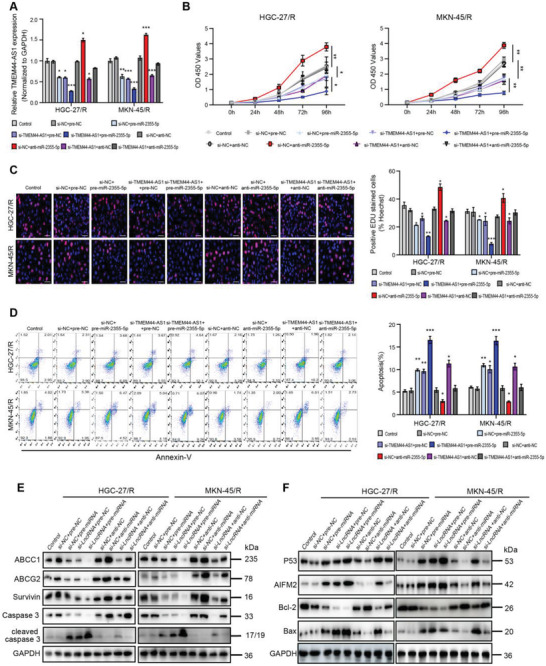
MiR‐2355‐5p participates in effects on 5‐FU resistance associated with TMEM44‐AS1 knockdown in cells with 5‐FU resistance. A) RT‐qPCR was used to detect the expression of TMEM44‐AS1 after infection with si‐NC + pre‐NC, si‐NC + pre‐miR‐2355‐5p, si‐TMEM44‐AS1+pre‐NC, si‐TMEM44‐AS1+pre‐miR‐2355‐5p, si‐NC+anti‐NC, si‐NC+anti‐miR‐2355‐5p, si‐TMEM44‐AS1+anti‐NC, or si‐TMEM44‐AS1+anti‐miR‐2355‐5p. B) 5‐FU (5 *μ*g mL^−1^) was added to cell medium, cell proliferation ability in different HGC‐27/R and MKN‐45/R cell lines experimental groups were detected by CCK8 assay. The relative proliferative rates at different time points were normalized to 0 h. C) Cell proliferation ability in different HGC‐27/R and MKN‐45/R cell lines experimental groups were detected by EdU assay. Scale bar, 20 µm. D) Cell apoptosis rate was detected for both resistant cells by flow cytometry analysis. E) Protein expression levels of ABCC1 and ABCG2, Survivin, Caspase3, cleaved caspase3, and GAPDH were detected by western blotting. F) Protein expression levels of p53, AIFM2, Bcl‐2, Bax, and GAPDH in HGC‐27/R and MKN‐45/R cells. 5 µg mL^−1^ 5‐FU was used to treat cells for a 48‐h period before EdU assay, flow cytometric analysis, and protein extraction. Data are mean ± SD, *n* = 3. *P < 0.05, **P < 0.01, ***P < 0.001.

For investigating the effect of TMEM44‐AS1 on promoting 5‐FU resistance in GC through its sponging effect on miR‐2355‐5p, this study conducted rescue assays using pre‐miR‐2355‐5p and anti‐miR‐2355‐5p following si‐TMEM44‐AS1 treatment. 5‐FU‐resistant cell lines were classified as the following nine groups: a control, si‐NC + pre‐NC, si‐NC + pre‐miR‐2355‐5p, si‐TMEM44‐AS1 + pre‐NC, si‐TMEM44‐AS1 + pre‐miR‐2355‐5p, si‐NC + anti‐NC, si‐NC + anti‐miR‐2355‐5p, si‐TMEM44‐AS1 + anti‐NC, as well as si‐TMEM44‐AS1 + anti‐miR‐2355‐5p groups. Then, TMEM44‐AS1 expression levels were determined in the nine groups using RT‐qPCR (**Figure** **5**A). Based on these results, TMEM44‐AS1 knockdown suppressed GC cell proliferation but enhanced their apoptosis; however, this effect could be rescued by application of anti‐miR‐2355‐5p and attenuated by pre‐miR‐2355‐5p administration (Figure 5B–D). Western blot results were consistent with the observed functional changes, i.e., si‐TMEM44‐AS1‐treated 5‐FU‐resistant cells displayed decreased ABCC1, ABCG2, Survivin, Caspase3, and Bcl‐2 levels and increased cleaved caspase‐3, P53, AIFM2, and Bax levels, and these changes could be rescued by the administration of anti‐miR‐2355‐5p and attenuated by pre‐miR‐2355‐5p treatment (Figure 5E,F). The above data implied that the effects on 5‐FU resistance associated with TMEM44‐AS1 knockdown in GC cells with 5‐FU resistance were mediated by miR‐2355‐5p.

### 2.6 PPP1R13L Was Involved in the TMEM44‐AS1/miR‐2355‐5p‐Dependent Impacts on GC Cell Resistance to 5‐FU

**Figure 6:**
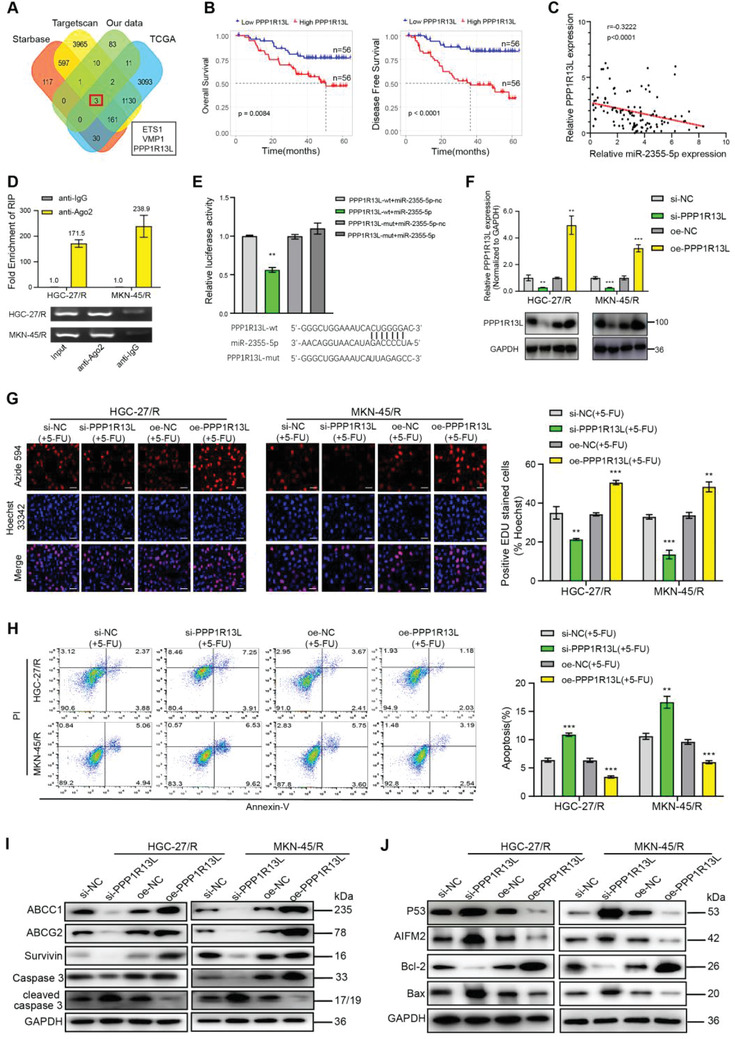
PPP1R13L was involved in the TMEM44‐AS1/miR‐2355‐5p‐dependent impacts on GC cell resistance to 5‐FU. A) Venn plot showing the four data sets. B) OS and DFS of GC patients (*n* = 112) with different PPP1R13L expression levels in their tumors. C) Linear regression analysis was done to each individual PPP1R13L and miR‐2355‐5p expression, *r* = −0.3222, *p* < 0.0001. D) RIP experiments were performed using the Ago2 antibody, and specific primers were used to detect the enrichment of PPP1R13L. E) Dual‐luciferase gene reporter assays were used to assess the binding sites of PPP1R13L and miR‐2355‐5p. The relative luciferase activities were restrained in the HEK‐293 T cells co‐transfected with PPP1R13L‐wt and miR‐2355‐5p. F) Transfection efficiency of si‐PPP1R13L and oe‐PPP1R13L was assessed by RT‐qPCR and WB assays. G) The proliferative abilities of HGC 27/R and MKN‐45/R cells subject to si‐NC, si‐PPP1R13L, oe‐NC, or oe‐PPP1R13L transfection were detected through EdU assay. Scale bar, 20 µm. H) The cell apoptosis rate was determined for the two GC cell lines with 5‐FU resistance through flow cytometry. I) ABCC1, ABCG2, Caspase3, cleaved caspase‐3, Survivin, and GAPDH protein expression was measured in HGC‐27/R and MKN‐45/R cells by western blotting. J) P53, AIFM2, Bcl‐2, Bax, and GAPDH protein levels within HGC‐27/R and MKN‐45/R cells. 5 µg mL^−1^ 5‐FU was used to treat cells for a 48‐h period before EdU assays, flow cytometric analysis, and protein extraction. Data are mean ± SD, *n* = 3. *P < 0.05, **P < 0.01, ***P < 0.001.

These above findings revealed that TMEM44‐AS1 and miR‐2355‐5p exert marked impacts on GC resistance to 5‐FU. To then predict miR‐2355‐5p target genes, we used two bioinformatics databases (starBase and TargetScan) in combination with TCGA and the genes found to be upregulated along with TMEM44‐AS1 in our sequencing results and identified *ETS1*, *VMP1*, and *PPP1R13L* as putative targets of miR‐2355‐5p (**Figure** **6**A). Next, we performed a survival analysis using tumor tissue specimens from the 112 GC patients and found that, among the three genes, only *PPP1R13L* showed a significant correlation with both OS and DFS in these patients (Figure 6B). Additionally, employing starBase, *PPP1R13L* level was negatively correlated with miR‐2355‐5p level within GC (Figure S11, Supporting Information). Furthermore, *PPP1R13L* levels within GC cells and patient samples were also analyzed, and *PPP1R13L* was negatively correlated with miR‐2355‐5p, but positively correlated with TMEM44‐AS1 (Figure 6C and Figure S12, Supporting Information). We then performed a RIP assay with AGO2 to determine whether PPP1R13L functions as a miRNA sponge, and found that PPP1R13L was enriched with AGO2 within two cell types with 5‐FU resistance (Figure 6D). As revealed by dual‐luciferase gene reporter assay, significantly decreased luciferase activity was detected when miR‐2355‐5p was overexpressed in the PPP1R13L‐wt group, while no effect was observed in the PPP1R13L‐mut group (Figure 6E). Subsequently, the PPP1R13L protein and mRNA expression reduced within si‐TMEM44‐AS1‐expressing cells when compared with those of cells treated with si‐NC, as revealed by WB and RT‐qPCR assays, respectively (Figure S13A, Supporting Information), and a similar effect was seen with pre‐miR‐2355‐5p treatment; in contrast, PPP1R13L levels were increased in cells expressing anti‐miR‐2355‐5p (Figure S13B, Supporting Information). In addition, the si‐TMEM44‐AS1‐mediated inhibition of PPP1R13L could be suppressed by co‐administration with anti‐miR‐2355‐5p and enhanced through pre‐miR‐2355‐5p co‐transfection (Figure S13C, Supporting Information). Once we had verified PPP1R13L as the miR‐2355‐5p target, we then knocked down PPP1R13L with siRNA and overexpressed PPP1R13L in cells resistant to 5‐FU (Figure 6F) and examined how altered PPP1R13L expression affected the 5‐FU‐resistant cell proliferation and apoptosis. According to Figure 6G, relative to controls, proliferation was remarkably reduced in cells of the si‐PPP1R13L group, while the opposite was observed in PPP1R13L‐overexpressing cells. Flow cytometric analysis demonstrated that the rate of apoptosis was increased with PPP1R13L depletion and decreased with PPP1R13L upregulation (Figure 6H). As revealed by WB assay, PPP1R13L downregulation inhibited ABCC1, ABCG2, Survivin, Caspase3, and Bcl‐2 expression and promoted that of cleaved caspase‐3, P53, AIFM2, and Bax expression; meanwhile, PPP1R13L upregulation exerted the opposite effects (Figure 6I,J). Combined, these data indicated that PPP1R13L was involved in the TMEM44‐AS1/miR‐2355‐5p‐mediated resistance to 5‐FU in GC cells.

### 2.7 CGE Nanoparticle‐Mediated TMEM44‐AS1 Silencing Synergistically Reversed 5‐FU Resistance

**Figure 7:**
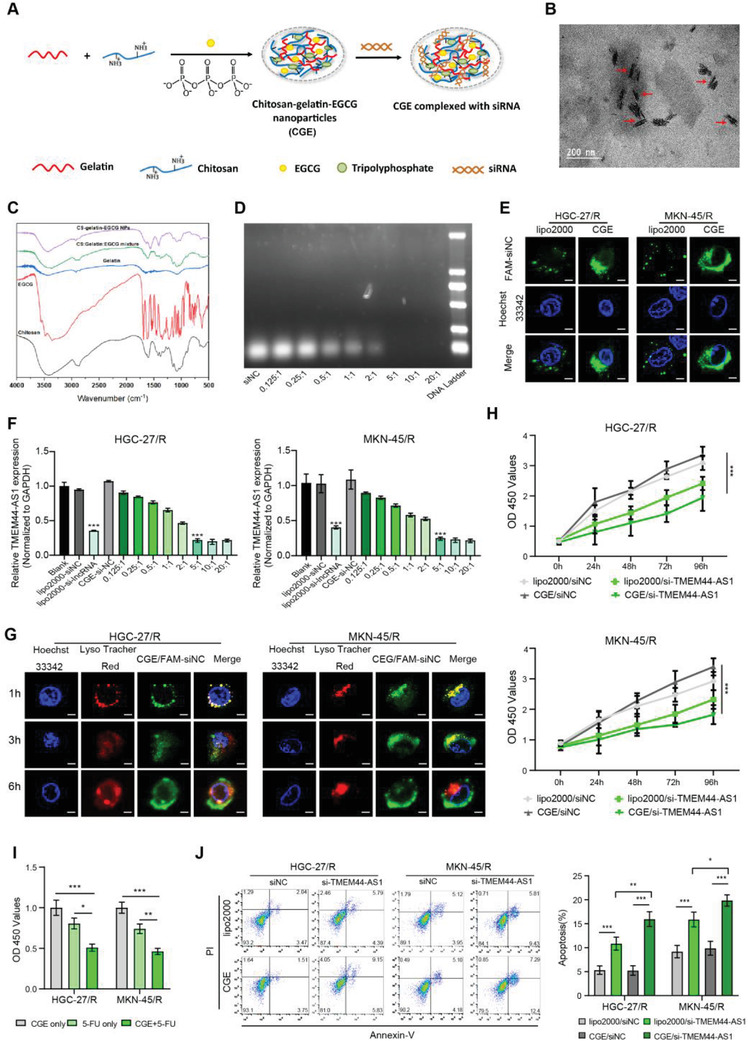
CGE nanoparticle‐mediated TMEM44‐AS1 silencing synergistically reversed 5‐FU resistance. A) Sketch map showing synthesis of CGE nanoparticles and siRNA loading. B) The shape of the CGE nanoparticles. C) FTIR spectrum of EGCG, chitosan, gelatin, mixture of EGCG, chitosan and gelatin, and CGE nanoparticles. D) Binding ability of CGE to siRNA at different mass ratios, assessed using gel electrophoresis. E) Confocal microscopy analysis of lipo2000/FAM‐siRNA or CGE/FAM‐siRNA distribution in HGC‐27/R and MKN‐45/R cells. Green: fluorescein‐labeled siRNA; blue: Hoechst 33342‐stained nuclei. Scale bar, 10 µm. F) Optimal ratio of CGE to siRNA was determined through RT‐qPCR. G) Representative confocal images of HGC‐27/R and MKN‐45/R cells incubated using CGE/FAM‐siNC under 37 °C for 1, 3, and 6 h. Hoechst 33342 (blue) was used to stain nuclei, LysoTracker Red (red) was used to stain endo/lysosomes, while FAM (green) was used to label siRNAs. Scale bar, 10 µm. H) After addition of 5‐FU (5 µg mL^−1^) into medium, proliferative ability of HGC‐27/R and MKN‐45/R cells subject to Lipo2000/si‐TMEM44‐AS1 or CGE/si‐TMEM44‐AS1 transfection was assessed through CCK‐8 assay. Proliferation rates at diverse time periods were calculated relative to that at 0 h. I) CCK‐8 assay was conducted to assess cell viability at 48 h post‐treatment with CGE+ 5‐FU, CGE only, or 5‐FU only. J) After 48 h of 5‐FU (5 µg mL^−1^) treatment, cell apoptosis rate was determined in cells treated with Lipo2000/si‐NC, lipo2000/si‐ TMEM44‐AS1, CGE/si‐NC, or CGE/si‐TMEM44‐AS1 by flow cytometry. Data are mean ± SD, *n* = 3. *P < 0.05, **P < 0.01, ***P < 0.001.

The above findings suggested that targeting TMEM44‐AS1 expression may be a promising strategy for reversing 5‐FU resistance. Here, we synthesized and employed CGE nanoparticles for the systemic delivery of si‐TMEM44‐AS1, and it may show synergistic effect on reversing 5‐FU resistance by the persistent TMEM44‐AS1 silencing (**Figure** **7**A). Figure 7B and Figure S14 (Supporting Information) displays size homogeneity and regular morphology of CGE nanoparticles. The average particle size was 141 ± 21 nm. The FTIR spectra of EGCG, chitosan, gelatin, mixture of EGCG, chitosan and gelatin, and CGE nanoparticles are presented in Figure 7C. Electrophoretic mobility shift assays in agarose gels were used for determining siRNA binding efficiency. In addition, CGE nanoparticles to siRNA ratio ranged from 0.125:1 to 20:1 (Figure 7D), showing the reduced siRNA mobility across the gel as CGE use amount increased. There was no siRNA band that exists at 5:1, suggesting the best CGE to siRNA ratio. Thereafter, we used CGE for delivering negative control siRNA (si‐NC) to HGC‐27/R and MKN‐45/R cells, following which we performed a CCK‐8 assay for assessing CGE cytotoxicity. CGE/si‐NC complexes at diverse doses were used to treat cells for 48 h. According to Figure S15 (Supporting Information), cell activity remained unaffected even at a ratio of 20:1. Next, we investigated the uptake behavior of the CGE with their siRNA cargo by tumor cells. We analyzed lipo2000/FAM‐siRNA or CGE/FAM‐siRNA complex adsorption via HGC‐27/R and MKN‐45/R cells with the fluorescein (FAM)‐labeled siRNA (FAM‐siRNA) through a confocal microscope. Microscopic observation of FAM‐labeled siRNA after 6 h of incubation showed that the CGE/FAM‐siRNA has better internalized efficiency by HGC‐27/R and MKN‐45/R cells than lipo2000/FAM‐siRNA (Figure 7E). Next, we validated the efficiency of TMEM44‐AS1 knockdown by the CGE. As confirmed by RT‐qPCR, the best CGE‐to‐siRNA ratio was 5:1 (Figure 7F). When siRNA entered cells, it must leave lysosomes. To investigate whether the siRNA delivered by the CGE could escape from lysosomes and achieve posttranscriptional gene silencing in the cytoplasm,^[^ ^21^ ^]^ CGE/siRNA was used to treat HGC‐27/R and MKN‐45/R cells for diverse durations, following which cellular localization of the CGE was determined. For investigating CGE/siRNA complex escape from lysosomes, we adopted LysoTracker to stain cells and then used a confocal microscope for monitoring. Nuclei were counterstained with Hoechst 33342. As shown in Figure 7G, following incubation for 1 h, the red (LysoTracker) and green (CGE/FAM‐si‐NC) fluorescence existed within cells simultaneously, indicative of the presence of FAM‐labeled siRNA in the lysosomes. With time, the overlap of green and red fluorescence decreased, particularly at 6 h, which indicated the escape of CGE/siRNA from lysosomes.

Having confirmed CGE/siRNA‐mediated gene silencing efficiency, we further analyzed the effect of CGE on synergistically reversing GC resistance to 5‐FU. First, 5‐FU‐resistant cells transfected with si‐TMEM44‐AS1 using CGE or Lipo2000, followed by 48 h of 5‐FU treatment were assessed for their proliferative and apoptotic ability. A CCK8 assay showed that cells co‐transfected with CGE and si‐TMEM44‐AS1 displayed weaker proliferative capacity than cells co‐transfected with either Lipo2000 and si‐TMEM44‐AS1 or CGE and si‐NC (Figure 7H). These results suggested that CGE can synergistically enhance 5‐FU‐mediated cytotoxicity. Next, we treated cells with CGE, 5‐FU, or CGE+ 5‐FU for a 48‐h period, as a result, CGE+ 5‐FU treatment group had decreased cell viability compared with the other two groups (Figure 7I). As revealed by flow cytometry, the apoptosis rate in CGE/si‐TMEM44‐AS1 treatment group increased compared with the other groups (Figure 7J). According to the above results, we speculated that weather the use of 5‐FU or siRNA can be reduced when CGE acts as a vector. We then detected the IC50 values and TMEM44‐AS1 expression levels of cells after transfecting with si‐TMEM44‐AS1 with lipo2000 or CGE, respectively. The experimental results show that the IC50 value of CGE/si‐TMEM44‐AS1 group is lower than that of lipo2000/si‐TMEM44‐AS1 group (Figure S16A, Supporting Information) and CGE‐mediated siRNA shows a better gene knockdown efficiency than lipo2000 (Figure S16B, Supporting Information). Therefore, it can be said that CGE‐mediated siRNA could reduce the dose of 5‐FU and siRNA.

### 2.8 CGE Nanoparticle‐Mediated TMEM44‐AS1 Silencing Enhanced 5‐FU Toxicity In Vivo

**Figure 8:**
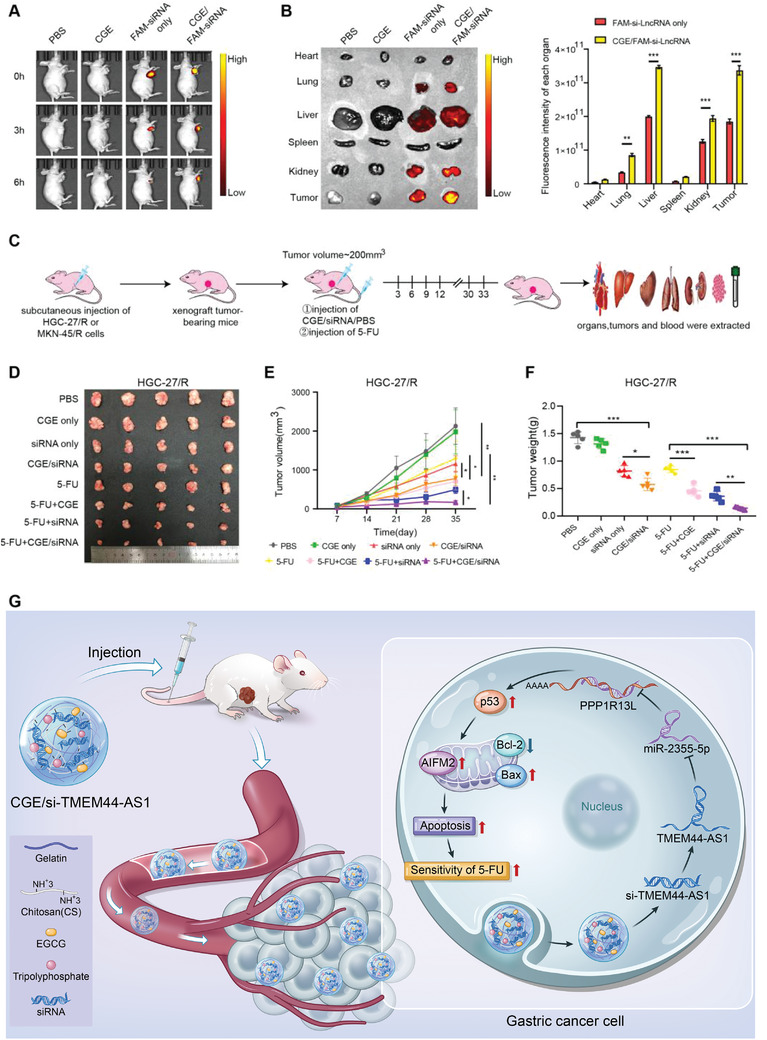
CGE nanoparticle‐mediated TMEM44‐AS1 silencing enhances 5‐FU toxicity in vivo. A) In vivo imaging of xenograft‐bearing mice after intratumoral injection of PBS,CGE,FAM‐si‐TMEM44‐AS1 only, or CGE/FAM‐si‐TMEM44‐AS1 (*n* = 3). B) In vivo imaging of xenograft‐bearing mice after tail vein injection of PBS, CGE, free FAM‐siTMEM44‐AS1, or CGE/FAM‐siTMEM44‐AS1 (*n* = 3). C) Sketch map showing tumor inoculation as well as diverse treatment of nude mice bearing tumor. D) Images of the collected subcutaneous xenograft tumors. E) Tumor volume in xenograft‐bearing nude mice was recorded weekly. F) Eventual weights of subcutaneous xenograft tumors. Data are mean ± SD, *n* = 3. *P < 0.05, **P < 0.01, ***P < 0.001. G) Schematic diagram of the mechanism underlying the role of CGE / si‐TMEM44‐AS1 complexes in increasing the sensitivity of gastric cancer cells to 5‐FU.

Finally, we sought to determine if CGE nanoparticles carrying si‐TMEM44‐AS1 could stay long in tumor or travel across bloodstream and arrive at the cancer sites, as well as their circulating stability. For this, GC tumor‐bearing mice were injected with PBS, FAM‐si‐TMEM44‐AS1 only, CGE only, or CGE/ FAM‐si‐TM4M44‐AS1 intratumor or via the tail vein. **Figure** **8**A shows that after intratumor injection with CGE/FAM‐si‐TMEM44‐AS1, more intense fluorescence at the subcutaneous tumor tissue was observed at 6 h compared with that of the FAM‐si‐TMEM44‐AS1 only group. As shown in Figure 8B, a greater accumulation of CGE/FAM‐si‐TMEM44‐AS1 complex was observed in the liver, heart, lungs, kidneys and spleen, most importantly, tumors, in xenograft mice in the CGE/FAM‐si‐TMEM44‐AS1 group than in those of the FAM‐si‐TMEM44‐AS1‐only group 6 h post‐injection via tail vein, which suggested the rapid clearance of siRNA via in vivo bloodstream without any nanoparticle coat. Based on this study, CGE/FAM‐si‐TMEM44‐AS1 complex shows favorable circulating stability, which is effectively delivered into cancer sites through in vivo injection via the tail vein. Accordingly, we divided HGC‐27/R and MKN‐45/R xenograft tumor‐bearing mice into the following eight groups: a PBS group, a CGE‐only group, a FAM‐si‐TMEM44‐AS1‐only group, a CGE/FAM‐si‐TMEM44‐AS1 group, a 5‐FU‐only group, a 5‐FU + CGE group, a 5‐FU + FAM‐si‐TMEM44‐AS1 group, and a 5‐FU + CGE/FAM‐si‐TMEM44‐AS1 group. When tumors had attained a volume of ≈200 mm^3^, the mice were treated with the above‐mentioned complexes every 3 d (Figure 8C). Tumor size was recorded weekly. At 35 d later, each animal was euthanized and the viscera, tumor, and blood samples were collected. Tumors in the CGE/FAM‐siRNA group showed small tumor size and weight compared with FAM‐siRNA‐only group; tumors within mice of the 5‐FU + CGE group showed small size and weight compared with 5‐FU‐only group; and mice of the 5‐FU + CGE/FAM‐siRNA group showed small size and weight compared with 5‐FU + FAM‐siRNA group (Figure 8D–F and Figure S17, Supporting Information). RT‐qPCR and immunohistochemical (IHC) staining of tumor sections were used to analyze the expression of TMEM44‐AS1 (Figure S18, Supporting Information) and PPP1R13L (Figure S19, Supporting Information) in the xenograft tumors. We further assessed the potential side‐effects of CGE nanoparticles in vivo. Blood serum analysis showed that IL‐1*β*, IFN‐*γ*, IL‐6, and TNF‐*α* contents were normal, as were several hematological indicators, such as urea, creatinine, alanine aminotransferase (ALT), and aspartate aminotransferase (AST) concentrations (Figure S20, Supporting Information). The liver, heart, kidney, lung, and spleen samples were subject to H&E staining, which showed no noticeable histological damage in these tissues (Figure S21, Supporting Information). Combined, the above results indicated that CGE have good biocompatibility and CGE‐mediated TMEM44‐AS1 silencing can enhance 5‐FU toxicity in vivo.

## 3 Discussion

Recently, substantial research works have been committed to elucidating the molecular mechanisms underlying chemoresistance in GC;^[^ ^22^ ^]^ however, these remain largely unclear. The present work first constructed the GC cell lines with 5‐FU resistance based on an in vivo model. Subsequently, we identified a novel lncRNA TMEM44‐AS1 as significantly increased within cells resistant to 5‐FU relative to respective sensitive cells through lncRNA sequencing analysis. Importantly, we discovered that high TMEM44‐AS1 level was in direct proportion to chemoresistance and dismal survival outcome for GC patients. Additionally, a variety of in vivo and in vitro assays showed that TMEM44‐AS1 competitively bound to and sponged miR‐2355‐5p. miR‐2355‐5p plays a role of the tumor suppressor within some cancers, like chondrosarcoma^[^ ^23^ ^]^ and bladder cancer.^[^ ^24^ ^]^ We further identified *PPP1R13L* as a miR‐2355‐5p target gene. PPP1R13L is a known inhibitor of P53 and takes an important position in the progression and chemoresistance of several cancers.^[^ ^25^ ^]^ High PPP1R13L expression results in the inhibition of the P53 signaling pathway, leading to reduced rates of apoptosis and increased drug resistance. Up to now, this is the first study reporting expression pattern of TMEM44‐AS1 as well as its regulation in GC.

The dysregulated expression of several lncRNAs, such as CRNDE, TINCR, and PDIA3P1, has been associated with chemotherapy resistance in several human cancers,^[^ ^26^ ^]^ highlighting that lncRNAs might represent attractive molecular targets for cancer treatment. Here, we demonstrated that TMEM44‐AS1 could promote tumor proliferation and decrease 5‐FU sensitivity in GC cells. Additionally, this study provided in vivo and in vitro evidence that combining TMEM44‐AS1 downregulation with 5‐FU treatment has a better efficacy at killing GC cells than either treatment alone. Consequently, evaluation of TMEM44‐AS1 expression was the effective approach to judgment high‐risk 5‐FU‐resistant GC cases, and clinicians could optimize clinical decisions based on the evaluation results, which might favor a more aggressive treatment regimen.

LncRNAs are known to act as miRNA sponges, competitively binding miRNAs and thereby regulating a variety of biological functions.^[^ ^27^ ^]^ Studies have remarkably proved that AGO2 serves as the indispensable part in RNA‐induced silencing complex.^[^ ^20^ ^]^ Only lncRNAs located in the cytoplasm can competitively bind miRNAs. In our work, TMEM44‐AS1 showed major cytoplasmic distribution, as verified by FISH. Employing starBase, miR‐2355‐5p was proved to be the possible microRNA target of TMEM44‐AS1, and confirmed this possibility through a series of experiments. Furthermore, PPP1R13L was identified to be miR‐2355‐5p's target gene. PPP1R13L is an evolutionarily conserved inhibitor of P53, a tumor suppressor playing a crucial role in suppressing growth, inhibiting progression of cell cycle, differentiation and apoptosis, while accelerating DNA repair. Our results suggest a model in which high TMEM44‐AS1 expression sponges miR‐2355‐5p, resulting in the upregulation of PPP1R13L, together with subsequent P53 pathway inhibition, with the consequent reduction in the rates of apoptosis and reduced 5‐FU sensitivity in GC.

RNAi therapeutics are suggested to be the potential novel type of pharmaceutical drugs, with siRNAs displaying the most promising silencing mechanism;^[^ ^28^ ^]^ however, effective and safe systemic siRNA delivery into target organs and tissues with a function of expressing target genes in humans remains a primary challenge for their application.^[^ ^29^ ^]^ The use of nanoparticles for this purpose represents a breakthrough in the field,^[^ ^30^ ^]^ and shows great promise as an option for disease treatment, including that of cancers. In the present study, we developed a novel nanosystem that can achieve efficient intracellular siRNA delivery and good anticancer effects. In vitro, we showed that the si‐TMEM44‐AS1 delivered via the CGE nanoparticles system had a higher gene silencing efficiency than via lipo2000 and could efficiently escape from lysosomes. In vivo, meanwhile, we demonstrated that this delivery system had few off‐target effects and favorable serum stability with the maintenance of silencing ability. SiRNAs delivery based on CGE nanoparticles can achieve effective and sustained silencing of TMEM44‐AS1 and synergistically reduce GC cell resistance to 5‐FU.

## 4 Conclusion

Collectively, this work identified prognostic influence of TMEM44‐AS1 in GC. Furthermore, TMEM44‐AS1 promotes GC cell proliferation but suppresses their apoptosis after 5‐FU treatment by sponging miR‐2355‐5p, leading to PPP1R13L up‐regulation and the consequent downregulation of the P53 signaling pathway. We also demonstrated that using CGE nanoparticles as carriers for si‐TMEM44‐AS1 can apparently silence TMEM44‐AS1 expression and reduce the 5‐FU resistance in GC cells. Additionally, systemic si‐TMEM44‐AS1 delivery using the CGE nanoparticles system can promote 5‐FU sensitivity in GC cells both in vivo and in vitro. This novel siRNA nanocarrier delivery system developed in this study provides a meaningful reference for treating chemotherapeutic‐resistant GC cases.

## 5 Experimental Section

### Patient and Tissue Specimens

In the present work, the use of patient samples gained approval from the Committees for Ethical Review of Research at Sun Yat‐Sen University (Guangzhou, China) ([2021]067). This study recruited a total of 112 GC cases at stage II–III receiving surgery at Sun Yat‐Sen University (Guangzhou, China) from January 2010 to December 2011. All the patients enrolled did not receive any treatment before surgery or 5‐FU‐based adjuvant chemotherapy after operation, namely, a XELOX regimen (capecitabine + oxaliplatin), a SOX regimen (tegafur + oxaliplatin), a FOLFOX regimen (oxaliplatin + CF + 5‐FU), tegafur, or capecitabine only. The clinicopathological characteristics and TMEM44‐AS1 expression level of these patients are shown in Table S1 (Supporting Information). Each case received regular follows‐up, and recurrence and/or metastasis were recorded. This study isolated total tissue RNA by adopting Trizol reagent (Invitrogen, Carlsbad, CA, USA).

Additionally, 60 GC cases at stage IV were also enrolled from January 2008 to December 2013 for analysis. They were administered fluorouracil‐based chemotherapy regimens (FOLFOX, XELOX, EOX, or SOX). Every six weeks, CT examination and tumor marker level were used to evaluate the patient's response to chemotherapy

### Overall Survival (OS) and Disease Free Survival (DFS)

In this study, OS indicated the duration between surgery date and all‐cause death date. DFS indicated the duration between surgery date and tumor relapse or mortality due to tumor progression.

### Cell Lines

HGC‐27 and MKN‐45 human GC cell lines, together with the HEK‐293 T human embryonic kidney cell line were obtained from the Chinese Academy of Sciences. HGC‐27 and MKN‐45 were cultured in RPMI 1640 medium (GIBCO BRL, Grand Island, NY) added with 10% FBS (PAN‐Seratech, Germany) as well as 1% penicillin/streptomycin (Invitrogen, Carlsbad, CA). Additionally, the HEK‐293 T cell line was cultivated with DMEM (Gibco) that contained 10% FBS as well as 1% penicillin/streptomycin. Cells were incubated under 5% CO_2_ and 37 °C conditions. 5‐FU was provided by Solarbio (Beijing, China). Immediately before use, 5‐FU was dissolved in physiological saline, yielding a 1 mg mL^−1^ stock solution.

### 5‐FU‐Resistant GC Cell Construction

The 5‐FU‐resistant GC cell lines were constructed according to the methods as previously reported.^[^ ^26^, ^31^ ^]^ Nude mice were given subcutaneous inoculation of altogether 5 × 10^6^ HGC‐27 or MKN‐45 cells via right flanks. From day 7 post‐inoculation, tumor diameter and width were measured every 3 d until the xenograft volume reached 200 mm^3^. Tumor size was determined by the formula *V* = 0.5 × *L* × *W* ^2^. Each mouse was then given intraperitoneal injection of 10 mg kg^−1^ 5‐FU or 0.9% NaCl at intervals of 2 d for altogether two consecutive weeks and no drug treatment for following two weeks (one course). After one course of treatment, xenografts were collected and transplanted in nude mice, and these xenografts were treated with 5‐FU or 0.9% NaCl. Once these animals had received four courses of 5‐FU or 0.9% NaCl treatment, GC cells (HGC‐27/S, HGC‐27/R, MKN‐45/S, and MKN‐45/R) were isolated from xenografts and the resistance to 5‐FU was confirmed. The Institutional Animal Care and Use Committee of the First Affiliated Hospital, Sun Yat‐sen University approved this study protocols ([2021]067).

### CCK‐8 Assay

To calculate the IC50, cells (5000/well) were inoculated into the 96‐well plates that contained 100 µL cell culture medium. Different concentrations of 5‐FU (0, 0.1, 0.2, 0.5, 1, 2, 5, 10, 20, 50, and 100 µg mL^−1^) were added once cells had adhered to the plates, and incubation for 72 h. Then, CCK8 reagent (10 µL, Dojindo Laboratories, Kumamoto, Japan) was added to the cultured cells for 2 h. Finally, the absorbance of cells was detected by the microplate reader (Varioskan LUX, Thermo Scientific, USA) at 450 nm. This study determined the proliferation inhibition rate IC50 by (1−experimental group/control group) × 100%. The method used to calculate cell viability was the same as that used for IC50 determination, except that the concentration of 5‐FU is 5 µg mL^−1^. To assess cell proliferative ability, the OD450 was measured at 0, 24, 48, 72, and 96 h after adding 5‐FU (5 µg mL^−1^). The rest of the procedures were as mentioned above.

### 5‐Ethynyl‐2′‐Deoxyuridine (EdU) Assay

2 × 10^5^ cells were inoculated on cover glasses (24 mm × 24 mm; CITOTEST, Jiangsu, China) placed at the bottom of wells of six‐well plates and cultured in medium containing 5‐FU (5 µg mL^−1^) in an incubator. After 48 h of treatment, 10 × 10^−3^ m EdU (Beyotime) was added to label cells for a 2‐h period, followed by 15 min of 4% paraformaldehyde (Beyotime) fixation, and another 15 min of cell treatment using PBS that contained 0.3% Triton X‐100 (AIDISHENG, Jiangsu, China). Later, cells were rinsed by PBS that contained 3% bovine serum albumin (BSA; Biofroxx, Germany), click additive solution (0.5 mL) was added into every well, then cells were later incubated for a 30‐min period under ambient temperature in dark, and counterstained with 1× Hoechst 33342 for 10 min. Cells positive for EdU and Hoechst 33342 staining were imaged by the automatic inverted fluorescence microscope (IX83, Olympus, Japan).

### Apoptosis Assay

Cell apoptosis was assessed by an Annexin V‐Alexa Fluor 647 or 488/propidium iodide (PI) Apoptosis Detection Kit (4A BIOTECH, Beijing, China). Collecting cell culture medium into a centrifuge tube and digesting cells with EDTA‐free trypsin, and the cell culture medium was again added to the cells. After centrifugation, PBS was used to wash the cells, which were resuspended in 1× binding buffer, with the cell density being adjusted to about 1–5 × 10^6^ mL^−1^. Annexin V‐Alexa Fluor 647 or 488 (5 µL) was added to the cells, and then cultured in the dark at room temperature for 5 min. Thereafter, the PI (10 µL) was placed in PBS to the flow cytometric tube. Ultrahigh‐speed flow cytometry (Invitrogen Attune NxT, ThermoFisher) was used to detect cell apoptosis. This study used FlowJo_V10 software for analyzing flow cytometry data.

### Western Blotting Assay

RIPA buffer (ThermoFisher) that contained a protease inhibitor (Beyotime) was used to lyse treated GC cells on ice. Later, cell lysates were incubated for a 30‐min period on ice, followed by 10 min of centrifugation at 12 000× *g* and 4 °C. Subsequently, supernatants were collected and the protein content was measured with the BCA protein assay kit (ThermoFisher). SDS‐PAGE was performed to separate cell proteins, which were later transported to PVDF membranes (0.22 µm; Millipore, MA, USA). Afterward, skimmed milk was used to block membranes for a 1‐h period under ambient temperature, followed by incubation overnight using primary antibodies targeting ABCC1 (1:1000; #72202S), ABCG2 (1:1000; # 42078T), Survivin (1:1000; #2808T), Caspase‐3(1:1000; #9664), cleaved caspase‐3 (1:1.000; #9664), Bcl‐2 (1:1000; #15071T), Bax (1:1000; #14796) (all from Cell Signaling Technology, Danvers, MA), GAPDH (1:1000; #60004‐1‐Ig), P53 (1:1000; #10442‐1‐AP), AIFM2 (1:1000; #20886‐1‐AP), and PPP1R13L (1:1000; #51141‐1‐AP) (all from Proteintech Group, Chicago, IL) under 4 °C. The next day, Tris‐buffered saline that contained 0.1% Tween 20 (TBST) was adopted to wash PVDF membranes, followed by another 1 h of incubation using secondary antibody (1:10000; Proteintech) under ambient temperature and TBST rinsing. The Western blot substrate kit (Tanon, China) was used to detect the immunoreactivity with electrochemiluminescence detection (Amersham Imager 600; GE, USA).

### RNA Sequence Analysis

This study adopted Trizol reagent for collecting total RNA in 5‐FU‐resistant and sensitive GC cells. A cDNA library was established according to specific protocols. With regard to library quality control, fragment size distribution was evaluated by Agilent 2100 bioanalyzer. In addition, qRT‐PCR (TaqMan Probe) was conducted to quantify libraries. Later, the BGISEQ‐500/ MGISEQ‐2000 System (BGI‐Shenzhen, China) was used for pair‐end sequencing of those eligible libraries. “Limma” package in R (R x64 4.0.4) was also used for identifying differentially expressed genes (DEGs) among the sequencing results. The top 50 DEGs (FoldChange (FC)| ≥ 2 and *P*‐value ≤ 0.05) of the two groups were used to generate a heatmap in R. Funrich software (Funrich 3.1.3) was used to plot a Venn diagram.

### RT‐qPCR

Total cellular RNA was extracted with Trizol, quantified using a NanoPhotometer (IMPLEN, Germany), and reverse‐transcribed (500 ng) to synthesize cDNA. The 2× Color SYBR Green qPCR Master Mix (EZBioscience, Beijing, China) was adopted to performed qRT‐PCR on a QuantStudio 5 RT‐PCR System (ThermoFisher). Table S2 (Supporting Information) shows the primer sequences. GAPDH and U6 served as the references for mRNA/lncRNA and miRNA Expression, respectively.

### CGE/siRNA Preparation

Chitosan (0.3 g) was dissolved in 15 mL of acetic acid buffer (pH 5.5) to form a transparent solution. Then, gelatin (0.15 g) was dissolved into water (15 mL) under 50 °C and stirring. EGCG (0.02 g) was added to the gelatin solution under stirring until dissolved, and then this mixture was added to the chitosan solution under stirring. Then, 1.5 mL of 3 mg mL^−1^ sodium tripolyphosphate (STPP) was added into mixed solution drop by drop under ambient temperature with stirring for 3 h. Later, suspension containing crosslinked GCE nanoparticles was dialyzed against acetic buffer (pH 5.5) to remove unreacted STPP and free EGCG. The obtained CGE nanoparticles suspension was collected and preserved under 4 °C prior to analysis.

For siRNA loading, 10 µL of a solution containing the CGE nanoparticles was mixed with 100 pmol siRNA and incubated for a 1‐h period under ambient temperature. Gel electrophoresis was performed to evaluate siRNA loading efficiency.

### Animal Experiments

The 4‐5‐week‐old BALB/c female nude mice were provided by Vital River Laboratory Animal Technology Co., Ltd (Beijing, China) and randomly divided into 16 groups. Mice were given subcutaneous injection of an equal number (5×10^6^) of HGC‐27/R or MKN‐45/R cells via left armpits. This study determined tumor volume (*V*) by the formula *V* = 0.5 × L × W^2^. From day 7 post‐inoculation, tumor diameter and width were measured every 3 days until the xenograft volume had reached 200 mm^3^. Animals were given subsequent injection of PBS, FAM‐si‐TMEM44‐AS1 only, CGE, or CGE/FAM‐si‐TM4M44‐AS1 into tail vein (*n* = 3 in each group). Six hours post‐injection, these mice were euthanized, the organs isolated, and the complex biodistribution examined using Xenogen IVIS Lumina system (IVIS spectrum, PerkinElmer). Next, mice were administered equal volumes of PBS, CGE, siRNA, CGE/siRNA (half with and half without 5‐FU treatment [10 mg kg^−1^, intraperitoneal injection]) once every 3 d via the tail vein. Tumor volumes were measured weekly. After treatment for 35 d, all animals were subject to euthanasia, followed by collection of tumors, organs, and blood serum. All the animal studies gained approval from the Institutional Animal Care and Use Committee of the First Affiliated Hospital, Sun Yat‐sen University. The animal procedures were carried out in line with guidelines of the National Institutes of Health.

### IHC and Hematoxylin and Eosin (H&E) Staining

An anti‐PPP1R13L antibody (1:100 dilution, #51141‐1‐AP, Proteintech) was used for IHC. Then, 5‐µm paraffin sections were prepared to conduct H&E staining. The automatic inverted fluorescence microscope (Olympus IX83, Japan) was used to capture images and the IHC staining intensity was scored.

### Statistical Analysis

Each assay was conducted in triplicate and data were presented in a form of mean ± SD. SPSS 25.0, GraphPad Prism 8.0, or R (version 4.0.4) (https://www.r‐project.org/) was adopted for statistical analyses. Two groups were compared by unpaired, two‐tailed, Student's *t*‐tests, while three or more groups were compared by one‐way ANOVA with Bonferroni tests were used. The Pearson coefficients were calculated to assess the correlations. Survival analysis was performed using the Kaplan‐Meier curves, and statistical significance was calculated according to the log‐rank test. *P* ≤ 0.05 stood for statistical significance (*P < 0.05; **P < 0.01; ***P < 0.001).

## Conflict of Interest

The authors declare no conflict of interest.

## Author Contributions

M.Z., J.D., J.H., W.Y., and Z.Z. contributed equally to this work. M.Z., J.Q.D., and J.Q.H. designed this experiment. M.Z., J.Q.D., J.Q.H., W.Y., and Z.S.Z. carried out experiments. W.Y., Z.S.Z., K.B.H., Y.H.P., J.J.C., Y.P.L., and G.N.S. performed the data analysis. M.Z., S.Y., X.X.L., and J.X.Z. wrote and reviewed our manuscript. All authors read and approved the final manuscript.

## Supporting information

Supporting Information

## Data Availability

The data that support the findings of this study are available from the corresponding author upon reasonable request.
