# Supplementary material for: Chitosan‐Gelatin‐EGCG Nanoparticle‐Meditated LncRNA TMEM44‐AS1 Silencing to Activate the P53 Signaling Pathway for the Synergistic Reversal of 5‐FU Resistance in Gastric Cancer
Source: Adv Sci (Weinh). 2022 Jun 19;9(22):2105077. doi: 10.1002/advs.202105077 (PMC9353463; doi:10.1002/advs.202105077)
Supplement: Supplementary file 1 — Supporting Information [file ADVS-9-2105077-s001.pdf]

ADVANCED SCIENCE
Open Access

Supporting Information

for *Adv. Sci.*, DOI 10.1002/advs.202105077

Chitosan-Gelatin-EGCG Nanoparticle-Meditated LncRNA TMEM44-AS1 Silencing to Activate the P53 Signaling Pathway for the Synergistic Reversal of 5-FU Resistance in Gastric Cancer

Mi Zhou, Jiaqi Dong, Junqing Huang, Wen Ye, Zhousan Zheng, Kangbo Huang, Yihui Pan, Junjie Cen, Yanping Liang, Guannan Shu, Sheng Ye, Xuanxuan Lu* and Jiaxing Zhang**

Supplementary Materials for

Chitosan-gelatin-EGCG Nanoparticle-Meditated LncRNA TMEM44-AS1 Silencing to Activate the P53 Signaling Pathway for the Synergistic Reversal of 5-FU Resistance in Gastric Cancer

Mi Zhou, Jiaqi Dong, Junqing Huang, Wen Ye, Zhousan Zheng, Kangbo Huang, Yihui Pan, Junjie Cen, Yanping Liang, Guannan Shu, Sheng Ye*, Xuanxuan Lu*, Jiaxing Zhang*

* Correspondence: yesheng2@mail.sysu.edu.cn, luxuanxuan2@jnu.edu.cn, zhangjx25@mail.sysu.edu.cn

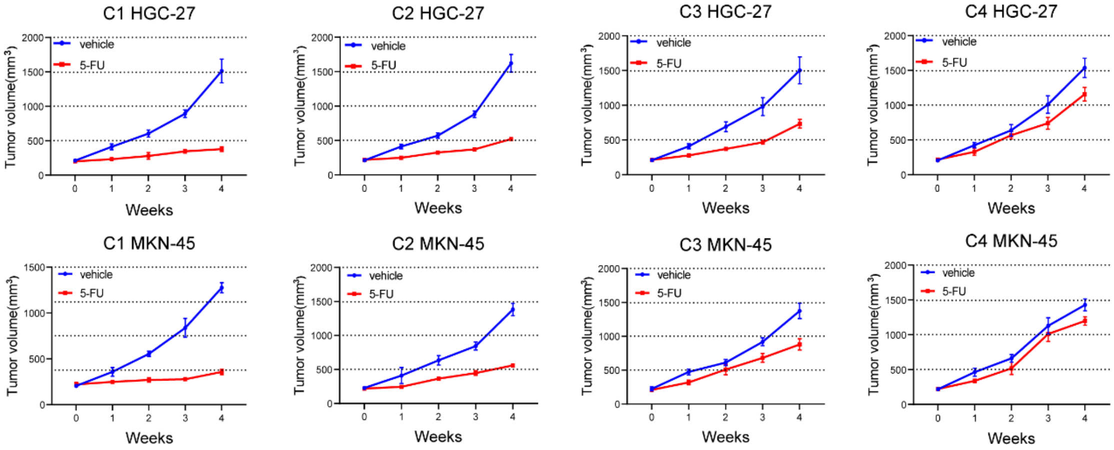

Figure S1. HGC-27 (upper) or MKN-45 (lower) xenografted nude mice were treated with vehicle or 5-FU (10 mg/kg) (n = 5 per group). Tumors were serially passaged in vivo. Tumor volumes are shown as mean ±SD.

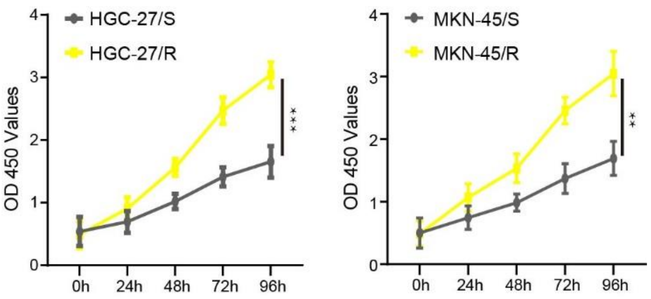

Figure S2. After the addition of 5 μg/ml 5-FU into medium, the proliferative ability of

sensitive and 5-FU-resistant cells was analyzed through CCK-8 assay. Relative proliferation rates at diverse time periods were calculated compared with that at 0 h.

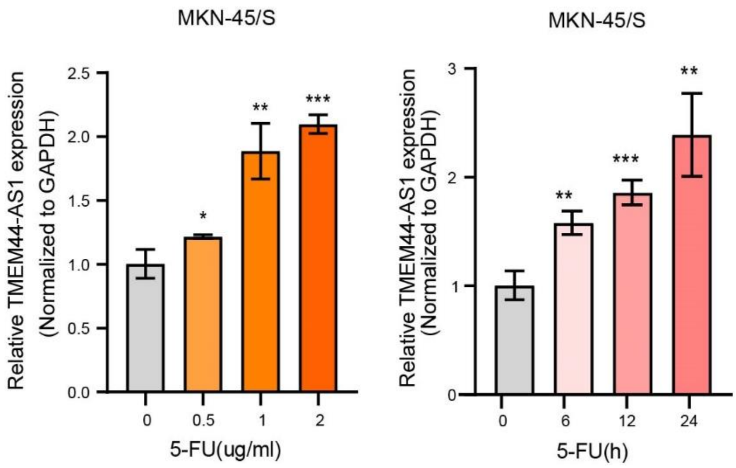

Figure S3. 5-FU treatment could induce up-regulation of TMEM44-AS1 in sensitive cell lines.

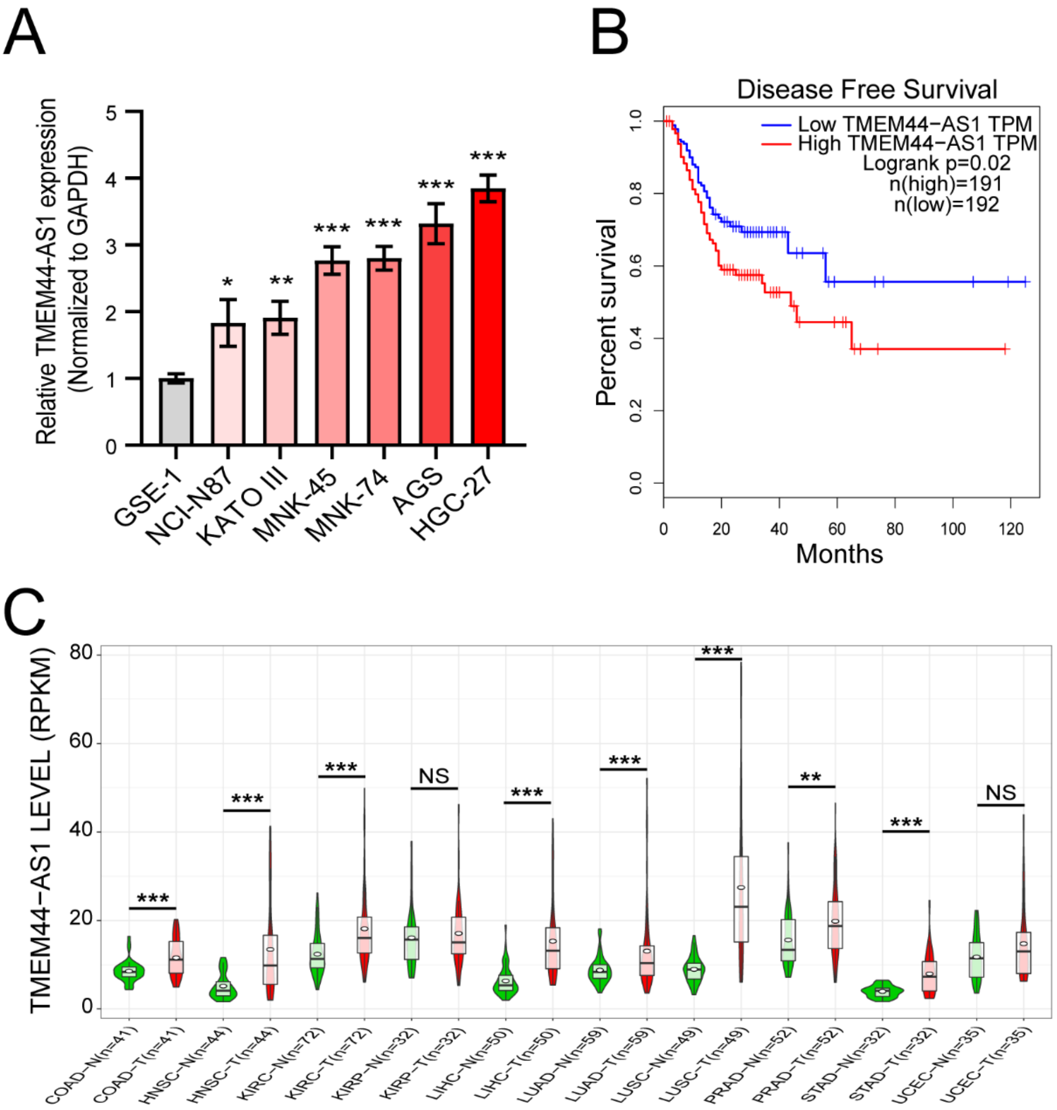

Figure S4. (A)The expression level of TMEM44-AS1 in GC cell lines and normal epithelial cells, measured by RT-qPCR. (B) The Cancer Genome Atlas (TCGA) database showed TMEM44-AS1 high expression is correlated with poor DFS. (C) TMEM44-AS1 up-regulation was common in different tumor types.

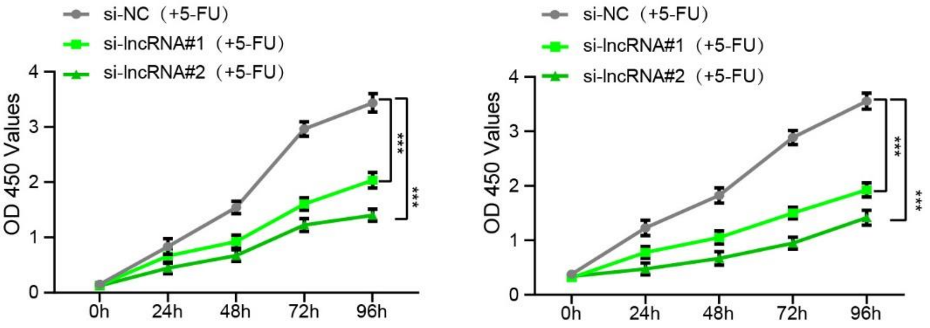

Figure S5. Addition of 5 μg/ml 5-FU into medium and the proliferative ability of HGC-27/R and MKN-45/R cells subject to TMEM44-AS1 siRNAs or si-NC

(negative control) transfection detected through CCK-8 assay. Proliferation rates at diverse time periods were calculated relative to that at 0 h.

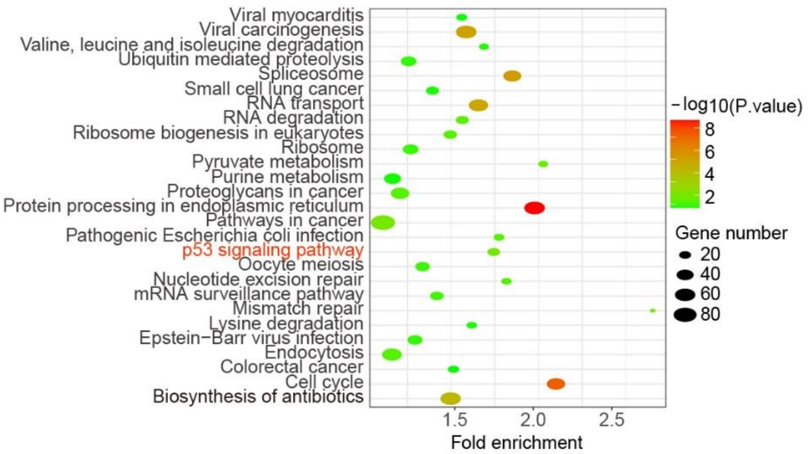

Figure S6. Protein-coding genes co-expressed with TMEM44-AS1 as determined by KEGG pathway analysis.

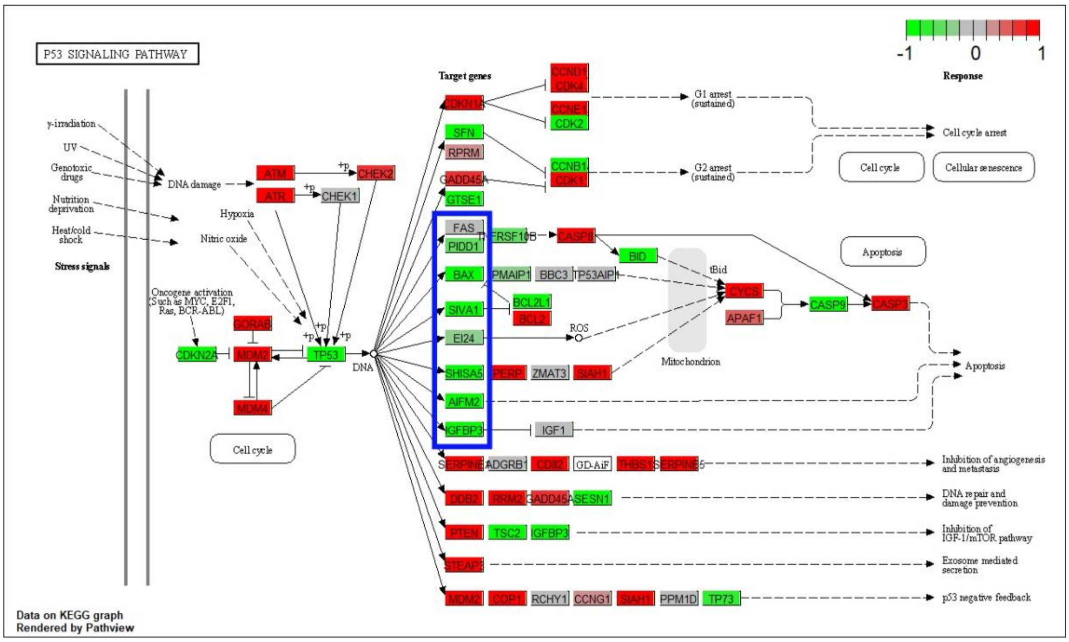

Figure S7. Visualizing genes in P53 pathway in our sequencing results.

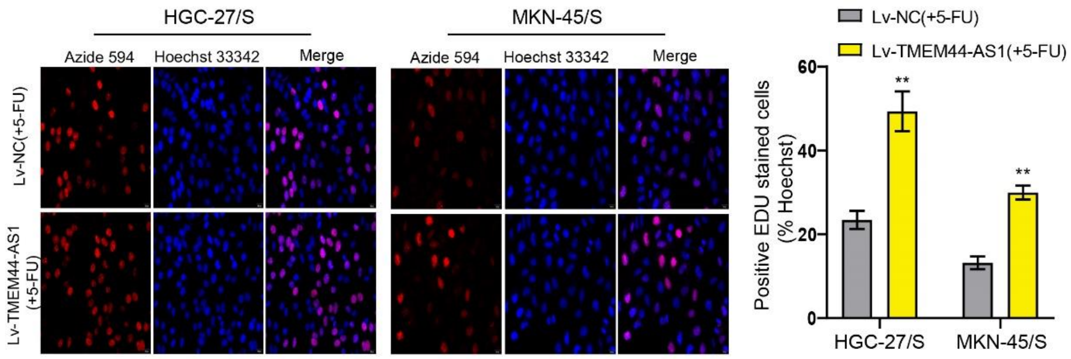

Figure S8. Cell proliferation ability of HGC-27/S and MKN-45/S transfected with Lv-TMEM44-AS1 or Lv-NC was detected by EdU assay.

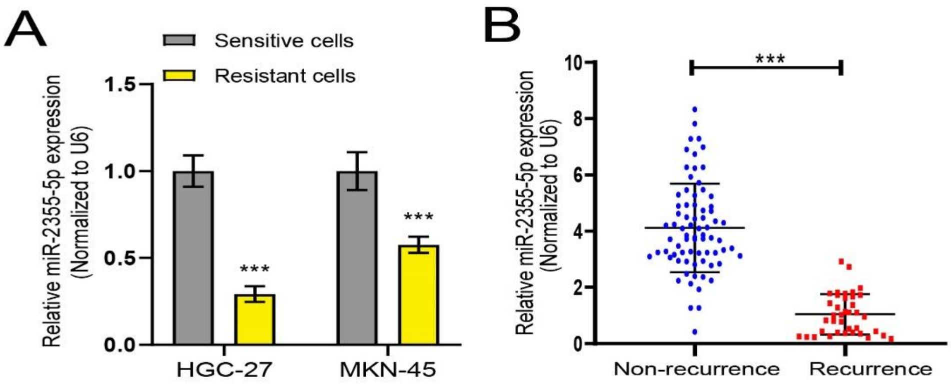

Figure S9. (A) RT-qPCR was used to detect the expression of miR-2355-5p in 5-FU sensitive and resistant cell lines. (B) Comparing differences in the expression levels of miR-2355-5p between GC tumor tissues from recurrent(n=38) and non-recurrent(n=74) patients.

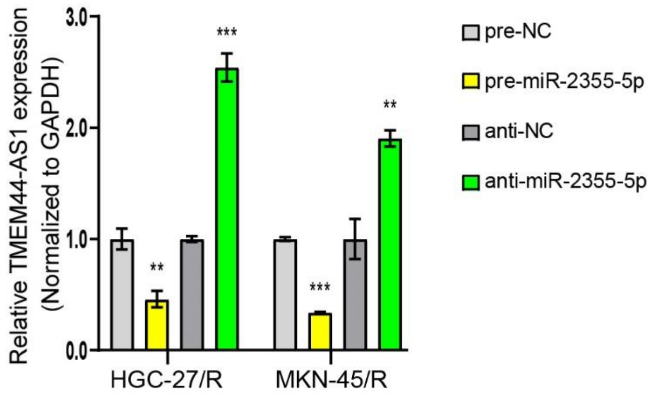

Figure S10. RT-qPCR was used to detect the expression of TMEM44-AS1 after miR-2355-5p level changes.

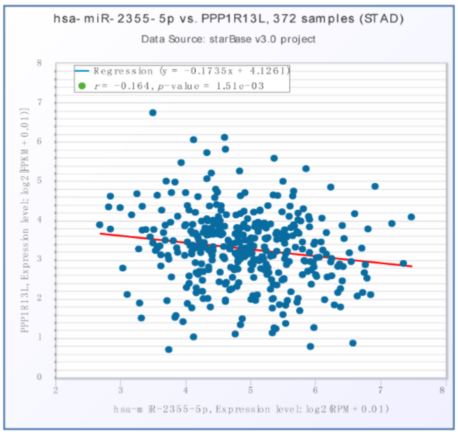

Figure S11. The negative correlation between PPP1R13L and miR-2355-5p in gastric cancer (Starbase database).

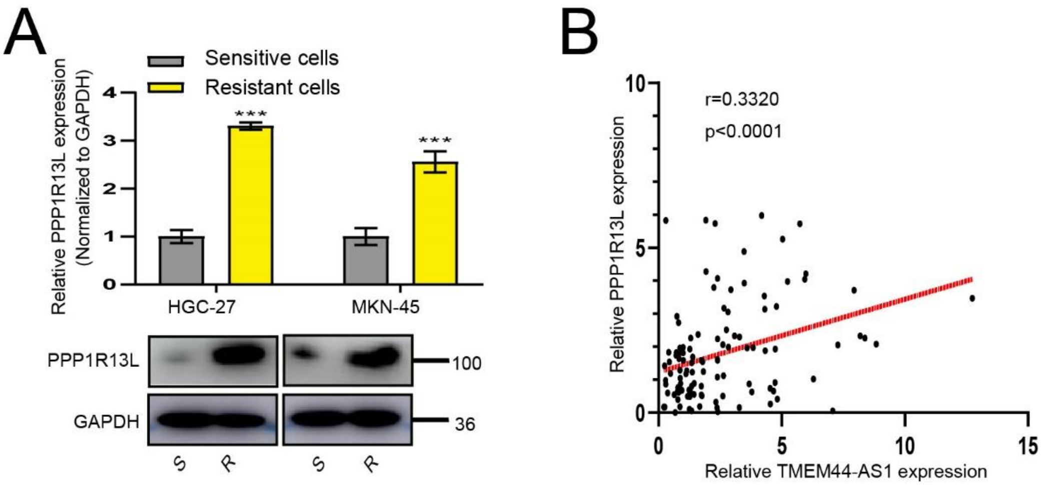

Figure S12. (A) Relative expression level of PPP1R13L in the HGC-27 and MKN-45 5-FU sensitive and resistance cell lines. (B) Linear regression analysis was done to each individual TMEM44-AS1 and PPP1R13L expression.

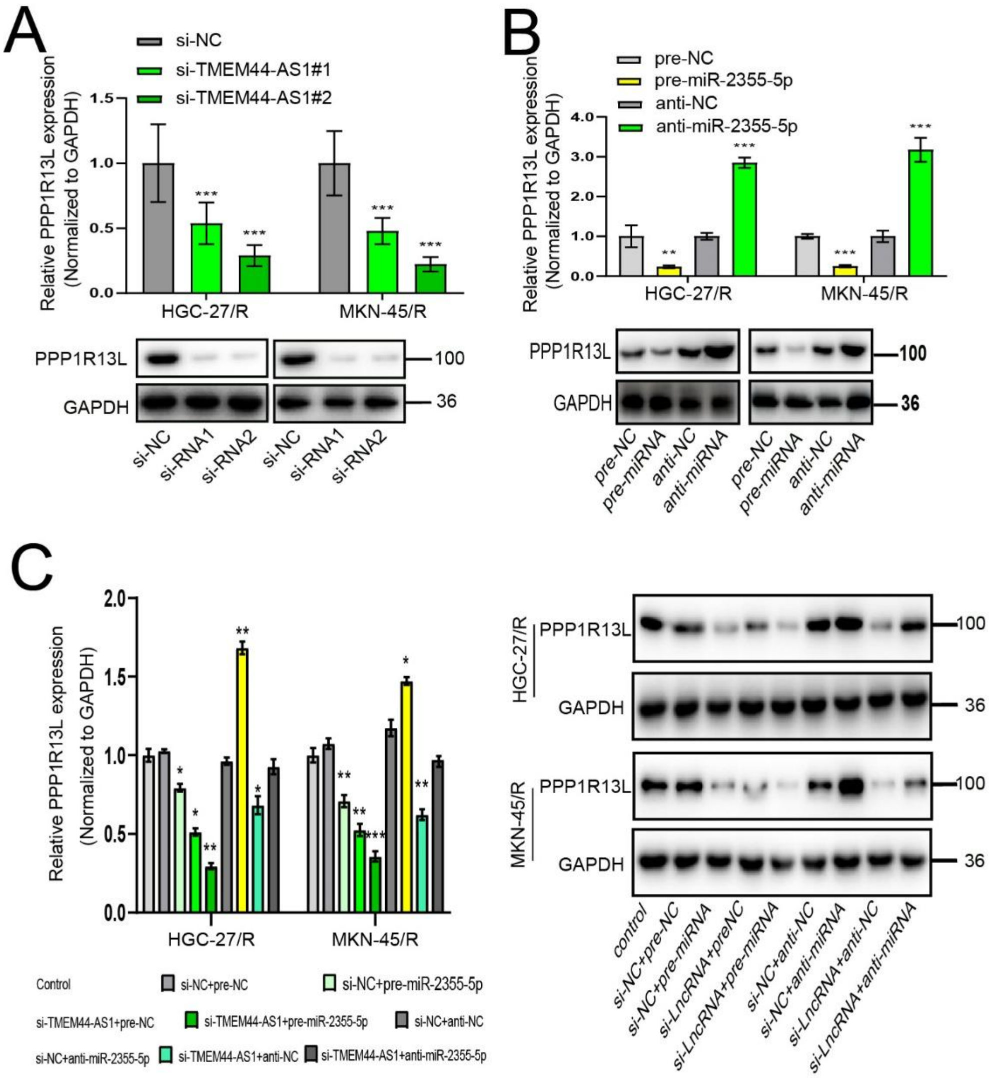

Figure S13. (A) RT-qPCR and WB was used to detect the expression of PPP1R13L after infection with si-NC, or si-TMEM44-AS1. (B) RT-qPCR and WB was used to detect the expression of PPP1R13L after infection with pre-NC, pre-miR-2355-5p, anti-NC,or anti-miR-2355-5p.(C) RT-qPCR and WB was used to detect the expression of PPP1R13L after infection with si-NC + pre-NC, si-NC + pre-miR-2355-5p, si-TMEM44-AS1+pre-NC, si-TMEM44-AS1+pre-miR-2355-5p, si-NC+anti-NC, si-NC+anti-miR-2355-5p, si-TMEM44-AS1+anti-NC, or si-TMEM44-AS1+anti-miR-2355-5p.

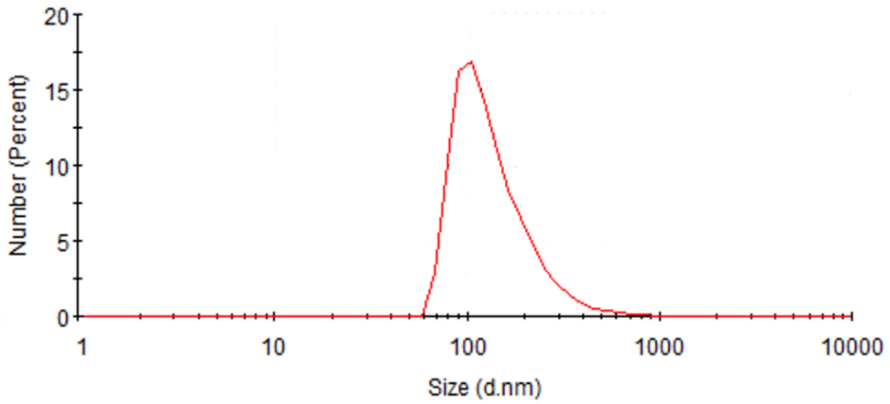

Figure S14. The size of the chitosan-gelatin-EGCG (CGE) nanoparticles.

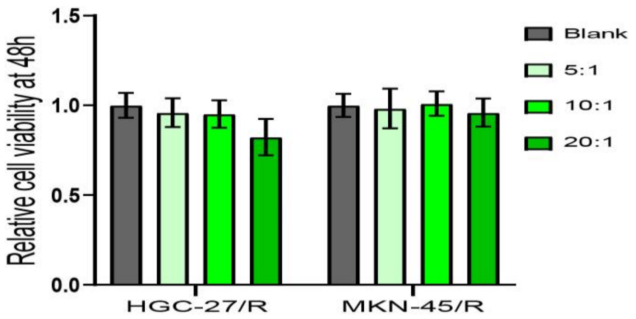

Figure S15. CCK8 was used to assess the cytotoxicity of CGE after these CGE were transfected into cells for 48 h. Cell viability was calculated as the percentage of viable cells relative to untreated control cells.

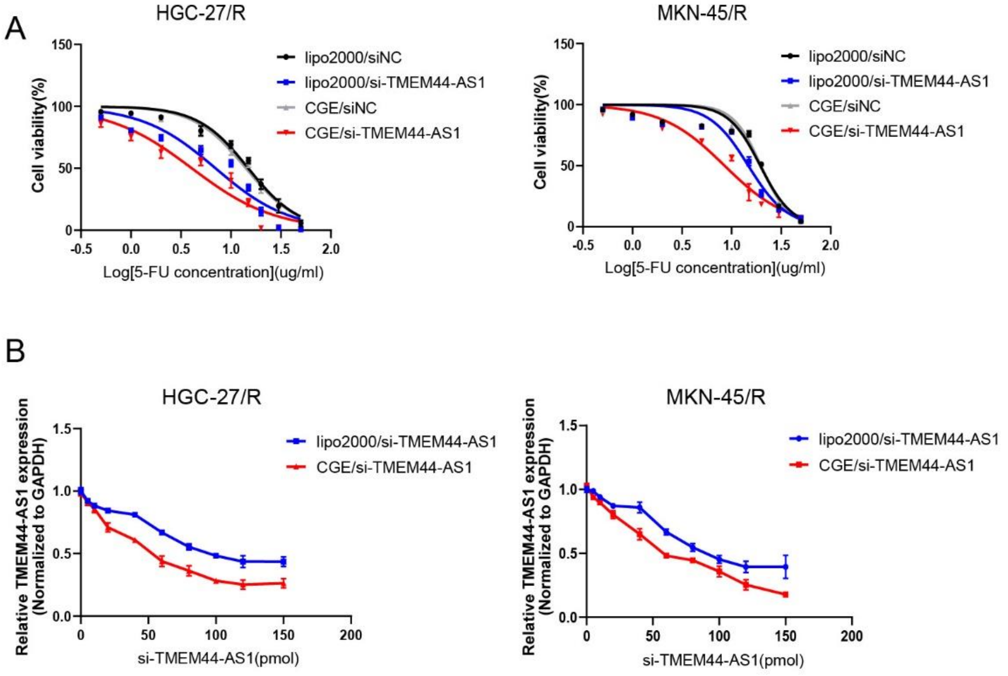

Figure S16. (A)The IC50 value was detected after added with 5-FU for 72h through CCK-8 assay. (B) TMEM44-AS1 silencing efficiency was measured through RT-qPCR after different doses of siRNA were transfected with lipo2000 or CGE as vectors, respectively.

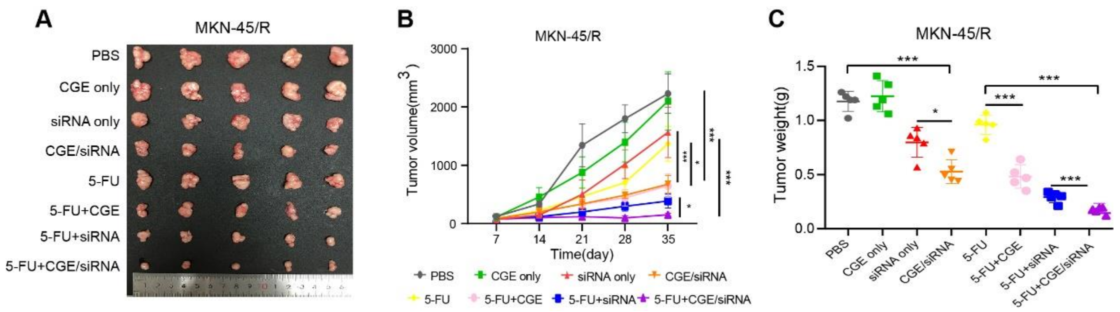

Figure S17. (A)Images of the collected subcutaneous xenograft tumors. (B) Tumor volume in xenograft-bearing nude mice was recorded weekly. (C) Eventual weights of subcutaneous xenograft tumors.

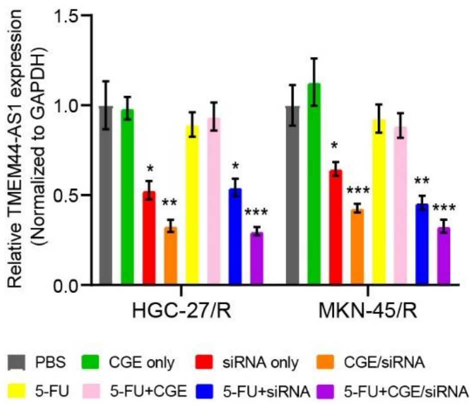

Figure S18. Tumor TMEM44-AS1 expression of different treatment groups were detected by RT-qPCR.

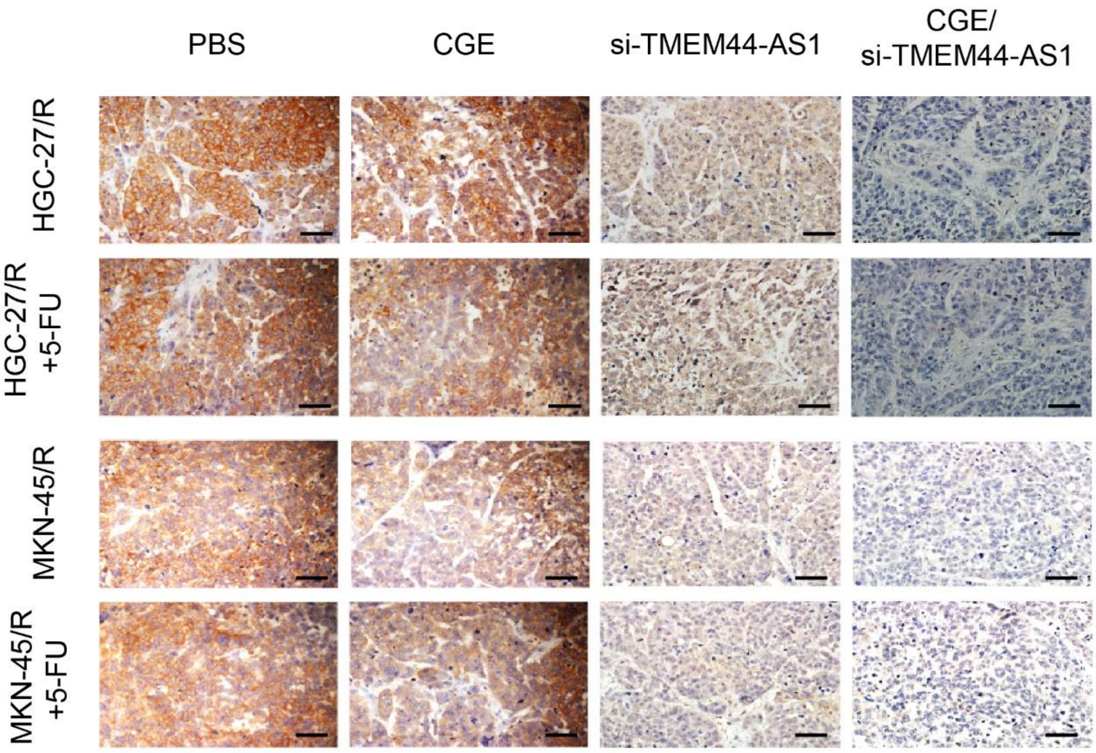

Figure S19. Immunohistochemical staining of tumors derived from subcutaneous xenografts. Scale bar, 100 μm.

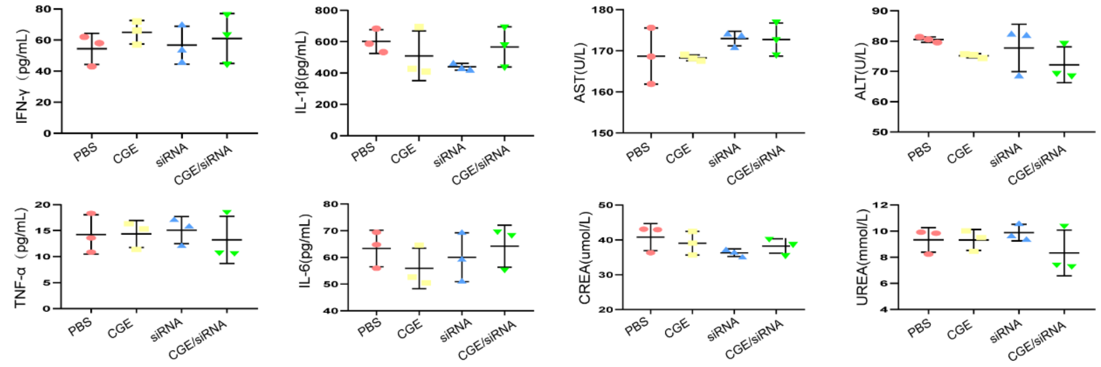

Figure S20. Serum levels of IFN-γ，IL-1β，TNF-α，IL-6,gaspartate

aminotransferase (AST), alanine aminotransferase (ALT), creatinine (CREA), and UREA after three consecutive injections of PBS, CGE, free siTMEM44-AS1 or CGE/siTMEM44-AS1.

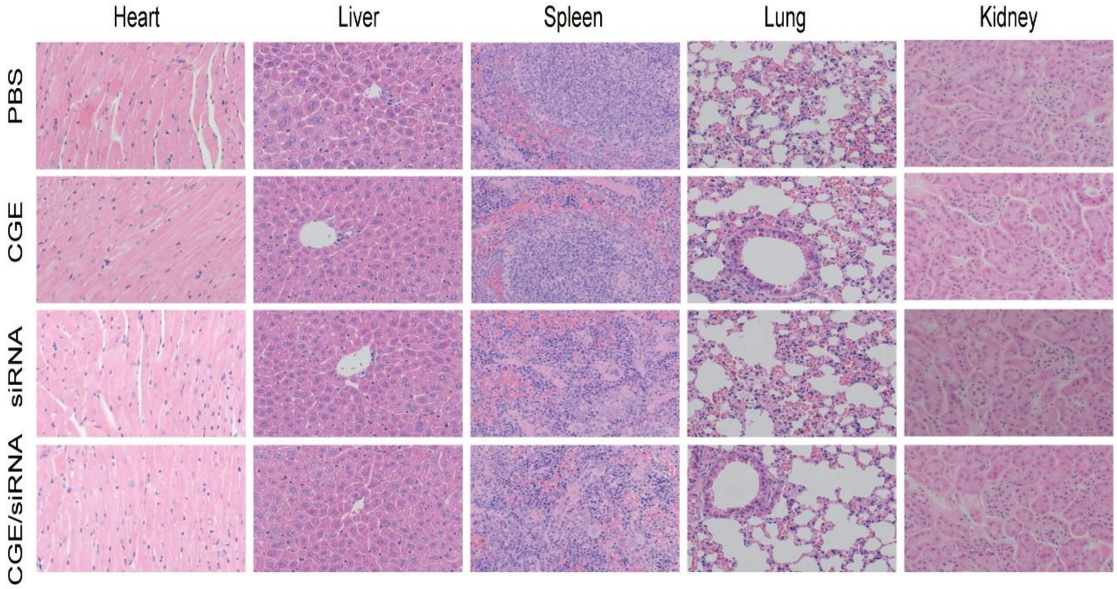

Figure S21. Histological section of the major organs after three consecutive injections of PBS, CGE, free siTMEM44-AS1 or CGE/siTMEM44-AS1.

Table S1. the clinicopathologic characteristics of the 112 GC patients who received surgery followed by adjuvant chemotherapy in this study.

Characteristics	No. of cases	TMEM44-AS1 expression		P value
		High	Low	
**Gender**				
Male	73	36	37	0.843
Female	39	20	19	
**Age(Years)**				
< 60	71	33	38	0.327
≥ 60	41	23	18	
**Clinical stage**				
II	38	8	30	<0.001
III	74	48	26	
**TNM status**				
II	61	22	39	0.001
**III**	51	34	17	
**Tumor size**				
< 5cm	65	29	36	0.18
≥ 5cm	47	27	20	

Table S2. Primers and DNA/RNA srquence used in this study

Primers	Sequence (5′-3′)
GAPDH F	GGAGCGAGATCCCTCCAAAAT
GAPDH R	GGCTGTTGTCATACTTCTCATGG
U6 F	CTCGCTTCGGCAGCACA
U6 R	AACGCTTCACGAATTTGCGT
TMEM44-AS1 F	CTGGGCAGACGATCTATTTGGA
TMEM44-AS1 R	TCGCTGGAACCTCTAAGGATTG
LINC01500 F	TGGCAACTATCACCACGGTC
LINC01500 R	TTTGATTGCCTGCATAGGCG
SERTAD3-AS1 F	CCGCGATTGGCTGGAGTC
SERTAD3-AS1 R	GGGAGCAATGGTGACGATCT
C11orf44 F	GTAGTCATGGGCCGCAGTAG
C11orf44 R	AGCATCCCGAGGAAAAGAGC
miR-545-5p F	TCAGTAAATGTTTATTAGATGA
miR-374a-3p F	CTTATCAGATTGTATTGTAATT
miR-2355-5p F	CCCAGATACAATGGACAA
PPP1R13L F	AGCCACACTGCTCTTCCTAAG
PPP1R13L R	TGTTTCATGGCCAGCGACT

Table S3. Correlation between the expression of TMEM44-AS1 and therapy response in GC patients (N = 60)

TMEM44-AS1 expression level				
**Therapy response**	**All cases**	**High expression**	**Low expression**	**P value**
CR+PR	30	10	20	p<0.001
SD+PD	30	23	7	

CR, complete response; PR, partial response; SD, stable disease; PD, progressive disease.

Supplemental Methods

Gene silencing or overexpression

Cells were seeded in 6-well plates and cultured overnight until they had attached to the plates. For gene silencing, after replacing the medium with fresh medium, the siRNAs were transfected into cells using Lipo2000 (Invitrogen) or CGE. ShRNAs were transfected using polybrene (Merck Millipore, Germany). After incubation for 8 h, the cells were washed with PBS, fresh medium was added, and the cells were incubated for another 48 h.

For gene overexpression, cells were seeded into 6-well plates and cultured overnight until they had attached to the plates. Overexpression plasmids were transfected into cells using Lipo3000 (Invitrogen), while lentiviral vectors for overexpression analysis were transfected into cells using polybrene. After incubation for 8 h, the cells were washed with PBS, fresh medium was added, followed by incubation for an additional 48 h. RT-qPCR was used to verify gene silencing or overexpression.

Rapid amplification of cDNA ends (RACE)

To determine the transcription initiation and termination sites for lncRNA TMEM44-AS1, 5'- and 3'-RACE assays were performed using Superscript IV Reverse Transcriptase (Invitrogen, Thermo Fisher Scientific) following the manufacturer's instructions. The sequences of the PCR-amplified 5′- and 3′-end fragments were confirmed by direct sequencing.

Fluorescence in situ hybridization (FISH)

Cy3-labeled TMEM44-AS1 and 18S probes were synthesized by GenePharma (Suzhou, China). A Fluorescent in Situ Hybridization Kit (GenePharma) was used to hybridize the probes to cells. Images were captured on an ultra-high-resolution laser confocal microscope (ZEISS LSM880 with Airyscan, Germany).

Nuclear and cytoplasmic separation

Nuclear and cytoplasmic fractions of cells were separated using a nucleus and cytoplasm separation kit (BB-36021-2; Bestbio, Shanghai, China). Approximately 5–10×10^6 cells were centrifuged for 2–3 min (4 °C, 500 × *g*) and then washed twice using cold PBS. Extract liquid A (200 μl) was added to the cell precipitate, the mixture was shaken on ice for 30 min, and then centrifugated (4 °C, 1,200 × *g*) for 5 min. The resulting supernatant was considered the cytoplasmic fraction and the precipitate the nuclear fraction.

Dual-luciferase reporter assays

Potential miR-2355-5p binding sites in TMEM44-AS1 and the *PPP1R13L* 3′-UTR were predicted using starBase and TargetScan (http://www.targetscan.org/). Wild-type and mutant reporter vectors were constructed by Genechem (pmirGLO, Shanghai, China). HEK-293T cells were seeded into 96-well plates, incubated overnight, and then transfected with luciferase reporter vectors using Lipo3000. Relative luciferase activities were measured using a Dual-Luciferase Reporter Assay Kit (Promega, USA) on a Varioskan LUX machine (Thermo Scientific) after 48 h of incubation. Firefly luciferase activity was normalized to that of *Renilla* (pRL-TK) luciferase and fold-changes in luciferase values calculated.

RIP assays

RIP assays were performed using the EZ-Magna RIP Kit (17-701, Merck Millipore) following the manufacturer’s instructions. Simply, cells were lysed with RIP lysis buffer. Then, A/G magnetic beads were used for immunoprecipitation of the antibody and the RNA binding protein. The magnetic beads were immobilized with a magnet and the unbound material was washed away. The RNA was subsequently collected and quantified by RT-qPCR. Input control and normal anti-IgG antibody control were also tested to ensure the accuracy of the detected signals from the protein-bound RNA.

Agarose gel electrophoresis

Agarose (HUAQISHENG, Guangzhou, China) gels (1.2%) were prepared using 1×

Tris-acetate (TAE) (Biosharp, Beijing, China) buffer. DNA was electrophoresed at 140 V for 30 min and the bands were visualized on an ultraviolet gel imaging system (UVP GelStudio PLUS touch, Germany).

Characterization of the CGE nanoparticle

The samples for characterization were prepared by diluting 1:500 (*w/v*) with deionized water. The particle size, polydispersity index (PDI), and zeta potential of the CGE nanoparticles were measured using a NanoSizer NanoZS analyzer (Malvern Instruments Ltd., Malvern, UK). All measurements were performed in triplicate. The morphological properties of the CGE nanoparticles were determined using a Tecnal-10 Transmission Electron Microscope (Philips, Netherlands) at an accelerating voltage of 200 kV. Samples were prepared by spreading CGE nanoparticles onto a carbon-coated microscope grid and drying at room temperature. Fourier-transform infrared spectra (FTIR) of EGCG, chitosan, gelatin, CGE nanoparticles, a mixture of EGCG, chitosan and gelatin were measured using a Spectrum GX spectrophotometer (PerkinElmer Inc., MA, USA) in the range of 400–4,000 cm^{-1}. EGCG concentration in the CGE nanoparticles was determined by measuring the free EGCG content in the dialysis medium using HPLC. Free EGCG was regarded as unloaded EGCG.

***In vitro* transfection and CGE/FAM-siRNA distribution**

Cells were seeded on cover glasses (24 mm × 24 mm; CITOTEST) placed at the bottom of wells in 6-well plates and cultured overnight until they had attached to the plates. The next day, 100 pmol FAM-siRNAs was added to the CGE followed by incubation for 1 h at room temperature. This mixture was then added to cells in the 6-well plate and incubated for another 6 h. Nuclei were counterstained with Hoechst 33342. The distribution of CGE/FAM-siRNA was observed by ultra-high resolution laser confocal microscopy (ZEISS LSM880 with Airyscan).

Lysosomal escape of siRNA

Cells were seeded in confocal dishes, incubated overnight, and then transfected with

CGE/FAM-siRNAs. After incubating for 1, 3, or 6 h, LysoTracker Red (Beyotime) was diluted with culture medium (1:15,000) and used to mark lysosomes for another 30 min. Hoechst 33342 was used for nuclear staining. The distribution of CGE/FAM-siRNAs and lysosomes was observed using an ultra-high-resolution laser confocal microscope (ZEISS LSM880 with Airyscan).

Analysis of hematological parameters

Aspartate aminotransferase (AST/GOT) (#C010-2-1), alanine aminotransferase (ALT/GPT) (#C009-2-1), BUN (#C013-1-1), creatine (#C011-1-1), IFN-γ (#H025-96T), IL-1β (#H002), TNF-α (#H052), and IL-6 (#H007) test kits were used for hematological analysis. The OD value was determined using a Varioskan LUX microplate reader.
